# Benchmark Structures and Conformational Landscapes of Amino Acids in the Gas Phase: A Joint Venture of Machine Learning, Quantum Chemistry, and Rotational Spectroscopy

**DOI:** 10.1021/acs.jctc.2c01143

**Published:** 2023-02-02

**Authors:** Vincenzo Barone, Marco Fusè, Federico Lazzari, Giordano Mancini

**Affiliations:** † Scuola Normale Superiore di Pisa, piazza dei Cavalieri 7, 56126 Pisa, Italy; ‡ DMMT-sede Europa, Universitá di Brescia, viale Europa 11, 25121 Brescia, Italy

## Abstract

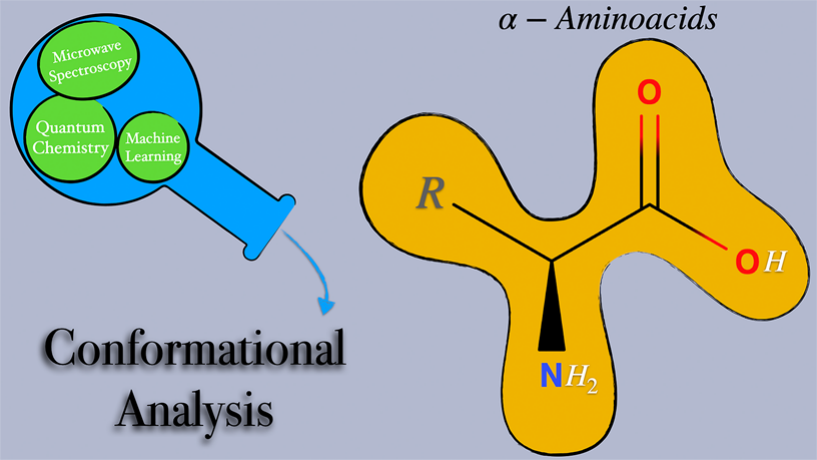

The accurate characterization of prototypical bricks of life can strongly benefit from the integration of high resolution spectroscopy and quantum mechanical computations. We have selected a number of representative amino acids (glycine, alanine, serine, cysteine, threonine, aspartic acid and asparagine) to validate a new computational setup rooted in quantum-chemical computations of increasing accuracy guided by machine learning tools. Together with low-lying energy minima, the barriers ruling their interconversion are evaluated in order to unravel possible fast relaxation paths. Vibrational and thermal effects are also included in order to estimate relative free energies at the temperature of interest in the experiment. The spectroscopic parameters of all the most stable conformers predicted by this computational strategy, which do not have low-energy relaxation paths available, closely match those of the species detected in microwave experiments. Together with their intrinsic interest, these accurate results represent ideal benchmarks for more approximate methods.

## 1 Introduction

Thanks to its high resolution and noninvasivity, gas-phase molecular spectroscopy has become the method of choice to investigate the role of intrinsic stereoelectronic effects in tuning the physical-chemical properties of biomolecule building blocks.^1,2^ In particular, the supersonic-jet expansion technique^3^ coupled to laser ablation^4^ is allowing the recording of gas-phase microwave (MW) spectra for these thermolabile compounds, which are usually characterized by high melting points.^5^ However, the fast relaxation of some structures to more stable counterparts in the presence of low energy barriers can bias any direct thermochemical interpretation of the results provided by this technique.^6,7^

Accurate quantum chemical (QC) computations can help to solve this kind of problem,^8,9^ but the effective exploration of flat potential energy surfaces (PESs) and the characterization of their stationary points for medium- to large-size flexible systems are still challenging for at least two different reasons. From the one side, the size of the systems prevents a brute force approach employing very accurate but very expensive state-of-the-art QC methodologies.^10−12^ From the other side, the very powerful local optimization techniques developed for semirigid systems are not effective for the exploration of rugged potential energy surfaces (PES) characterized by a huge number of energy minima possibly separated by low-energy barriers.^13,14^

This situation calls for an integrated computational approach employing QC models of increasing accuracy in the different steps of an exploration/exploitation strategy guided by machine learning (ML) tools.^13,15−17^ The effective strategy of this kind we have been developing in the past few years starts from a knowledge-based selection and constrained geometry optimizations of a limited number of conformers employing a fast semiempirical method.^14,18^ Next, an effective exploration of the whole conformational PES is performed by the same semiempirical method guided by a purposely tailored evolutionary algorithm with the aim of finding other low-lying minima.^13^ The results of this step are refined by hybrid and then double-hybrid density functionals,^19,20^ and possible relaxation paths between pairs of adjacent energy minima are identified.^16^ Once a panel of low-energy minima has been defined, accurate relative energies are computed by reduced-scaling composite methods.^21−26^ These results are integrated by zero point energies (ZPE) and thermal contributions to enthalpies and entropies employing anharmonic approaches rooted in the second order vibrational perturbation theory (VPT2)^27−34^ and proper treatment of hindered rotations.^35,36^ Finally, accurate spectroscopic parameters of the energy minima with nonnegligible populations under the experimental conditions of interest are computed.^37^ In the specific case of rotational spectroscopy, improved equilibrium rotational constants are obtained by refining the optimized geometries by a linear regression approach.^20,38^

Among the main biomolecule building blocks, natural α-amino acids, which exist exclusively in neutral form in the gas phase, represent a particularly appealing playground because their rich conformational landscape is tuned by the competition among different kinds of intramolecular hydrogen bonds. At the same time, MW results are available for several conformers of most natural α-amino acids,^39−50^ which represent very demanding benchmarks for the *a priori* prediction of structural and spectroscopic parameters. We have therefore selected the glycine and alanine prototypes together with a panel of α-amino acids with polar side chains (serine, threonine, cysteine, aspartic acid, and asparagine) with the aim of providing benchmark results allowing unbiased comparisons with experimental results. In fact, the current standards for the computation of MW parameters of biomolecule building blocks in the gas phase (see, e.g., refs (1, 6, 7, 39, and 51)) employ QC methods of limited accuracy, pay marginal attention to the geometrical parameters, and neglect vibrational corrections. However, these limitations hamper any *a priori* prediction of the spectroscopic outcome, allowing at most its *a posteriori* interpretation in terms of the agreement between experimental and computed spectroscopic parameters for a predefined number of conformers.

Based on these premises, the goal of the present study is to improve and validate a general strategy able to find all the conformers detectable in supersonic jet expansions taking also into account fast relaxation processes possibly leading to the disappearance of some low-lying species. Unbiased comparison with spectroscopic results is made possible by the accuracy of the computational results, which will be shown to provide mean unsigned errors (MUEs) within 20 MHz for rotational constants and 10 cm^–1^ for both relative energies and vibrational frequencies (entering zero point energies and thermal contributions to thermodynamic functions). Together with their intrinsic interest of the studied molecules, these results will provide also a reference set for more approximate methods and/or search techniques.

## 2 PES Exploration

**Figure 1:**
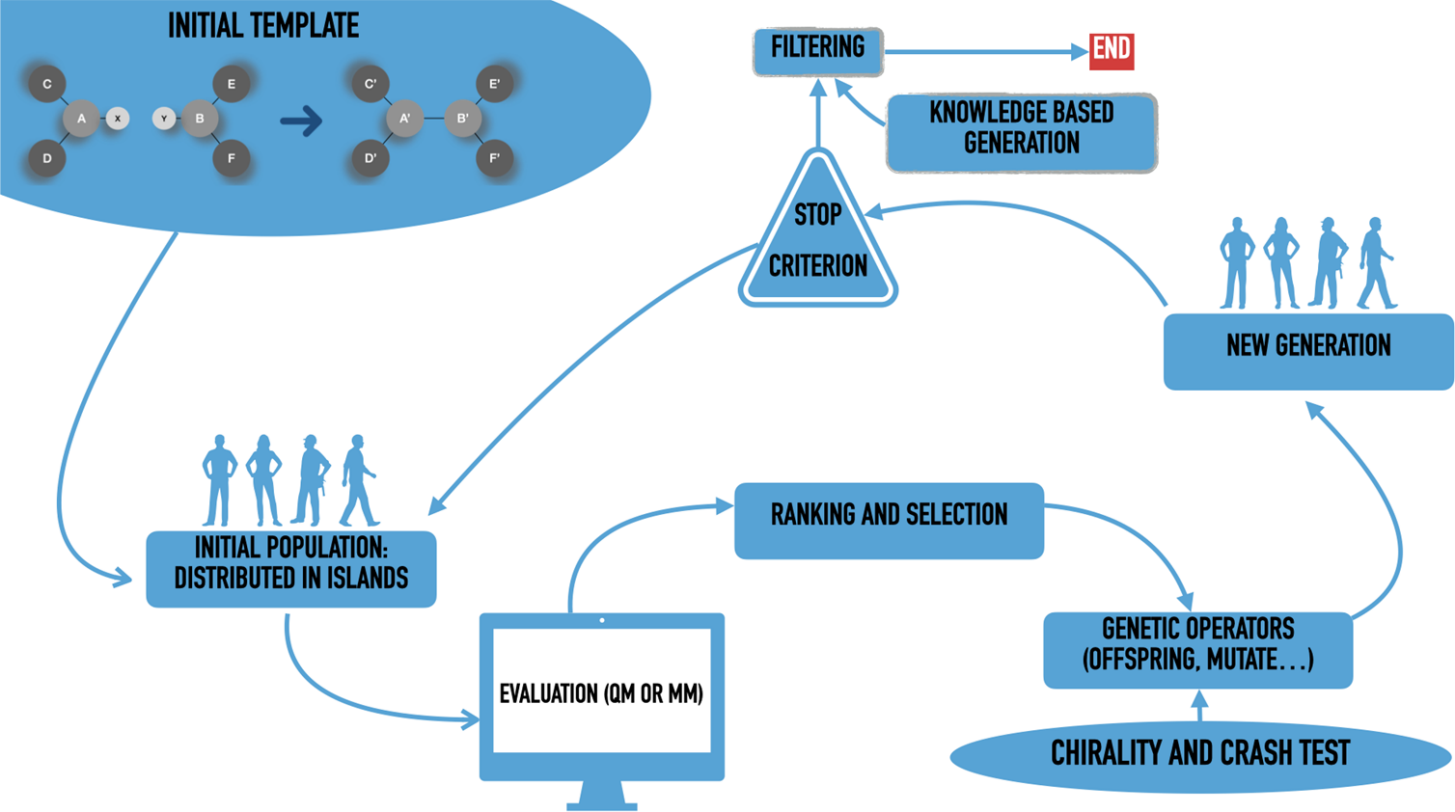
Flowchart of PES exploration. See main text for further details.

The general strategy for the exploration of conformational PESs is based on a continuous perception of molecular structures performed by the PROXIMA software,^52^ which is able to detect characteristic structural motifs and to separate soft (in the present context dihedral angles) and hard degrees of freedom. Then, a knowledge-based systematic search of soft degrees of freedom^14^ can be optionally performed, which produces a panel of guess structures (e.g., the 3^*n*^ staggered conformers generated by rotations around *n* nonterminal single bonds, which are not a part of cycles). The geometries of these candidates are next optimized using the fast GFN2-XTB semiempirical method,^18^ which has been selected because it tends to underestimate energy differences (i.e., to produce a too large set of candidates), which allows a safer use of energy thresholds for further processing. Next, a custom implementation of the island model evolutionary algorithm (IM-EA)^53^ is employed to produce other candidates starting from an initial population (*P*_0_) generated by the so-called Latin Hypercube stratified sampling^54^ in order to maximize the diversity of soft degrees of freedom. The chemical descriptor (fitness) of each structure is the relative electronic energy obtained by GFN2-XTB geometry optimizations of the stiff degrees of freedom. Improved populations are then built iteratively for a given number of cycles by applying, with predetermined probability, different genetic operators, namely, crossover (interpolation of the features of different related structures for creating new ones), mutation (change of one or more soft degrees of freedom with some stochastic rule), and selection (high chance for high fitness structures of propagating their features in the next cycles). In the IM-EA, the different operators act separately on disjoint regions of the conformational landscape (islands), which are mixed only at predefined intervals by a dedicated operator (migration). Furthermore, some of the best structures found in each cycle are directly transferred to the next cycle (the so-called Hall of Fame).^55^ All those choices are dictated by the high cost of evaluating the fitness of a new structure by constrained geometry optimizations. As a consequence, high fitness structures are worth being preserved in the population until some significantly improved structure is found. Typical values of the initial population, maximum number of cycles, and number of islands are 100, 50, and 4, which result in about 1000 constrained geometry optimizations for each run of the algorithm. In order to further increase the coverage of the conformational space, 4 runs with different initial populations are performed for each molecular system. The full set of parameters employed in the IM-EA algorithm is given in Table S1 of the Supporting Information (SI), while further details are given in refs (13 and 17). Figure 1 shows a schematic flowchart of the current version of the whole algorithm, which is available under the GPL3 license at https://github.com/tuthmose/IM_EA.

At the end of the whole exploration, low-energy conformers within a predefined energy range are selected from the panel of structures issued from IM-EA and, possibly, knowledge-based steps by eliminating too similar structures (in terms of rotational constants and root-mean-square deviations of heavy atom positions) and then performing single point energy evaluations at the B3LYP/jun-cc-pVDZ level,^56,57^ also including Grimme’s D3BJ dispersion corrections.^58^ In the following, this computational model will be referred to simply as B3. The choice of the specific functional is not critical in this step because it is used only for the selection of an initial panel of structures to be next refined at higher levels. The B3 model has been selected because it is routinely employed in the interpretation of MW studies and, more importantly, provides reasonable anharmonic corrections (vide infra).

In the next step, structures lying within a smaller energy range are optimized at the same level, and the surviving ones define the panel of candidates for the final structural refinement, which is performed employing the revDSD-PBEP86-D3BJ/jun-cc-pv(T+d)Z model^59−61^ (hereafter rDSD) for both geometry optimization and evaluation of harmonic force fields.^62^ The rDSD functional has been selected because several studies have shown that it provides excellent geometrical structures,^38^ dipole moments,^63^ spectroscopic parameters,^37^ noncovalent intermolecular interactions,^23,64^ and conformational landscapes.^10,65,66^

This composite strategy allows for strongly reducing the number of expensive geometry optimizations by hybrid and, especially, double-hybrid functionals without any loss of accuracy in the final results. The different energy thresholds depend on the system and the spectroscopic technique of interest. For the specific case of rotational spectroscopy, a conservative limit for the relative stability of detectable conformers is around 900 cm^–1^ (which corresponds to a relative population of about 1% at room temperature, where kT/hc = 207 cm^–1^).^1,16^ As a consequence, the typical thresholds for the acceptance of semiempirical structures, B3 geometry optimizations, and final rDSD refinement are 2500, 1500, and 1000 cm^–1^, respectively. These choices lead to about 100 B3 computations (including both single point and geometry optimizations) and no more than 20 rDSD geometry optimizations for each molecular system.

As mentioned in the Introduction, conformational relaxation can take place under the experimental conditions whenever the energy barriers ruling the interconversion are sufficiently low, with an upper limit of about 400 cm^–1^ being usually employed for discriminating in rotational spectroscopy of amino acids and related compounds.^6,7,67^ With the aim of unraveling fast conformational relaxations, we always perform relaxed torsional scans at the rDSD level in order to obtain preliminary information on low-energy interconversion paths. Next, after precise location of transition states (TSs) by full geometry optimizations, their nature is checked by computing Hessian matrices.

## 3 Relative Stabilities and Spectroscopic Parameters

The typical MUEs of rDSD bond lengths (0.003 Å) and valence angles (0.003 radians, i.e., 0.15°) observed in the large SE100 database^38^ are largely sufficient to obtain accurate relative electronic energies of different conformers by single-point energy evaluations using composite methods rooted in the coupled cluster (CC) ansatz.^68^ In particular, the CC model including single, double, and perturbative estimate of triple excitations (CCSD(T))^69^ is considered the gold standard for this kind of computations provided that complete basis set (CBS) extrapolation and core valence (CV) correlation are taken into the proper account. The key idea of the reduced cost Cheap scheme (ChS) is that, starting from frozen core (fc) CCSD(T) computations in conjunction with the cc-pVTZ basis set,^57^ CBS and CV terms can be computed with good accuracy and negligible additional cost employing second order Møller–Plesset perturbation theory (MP2).^70^ Several benchmarks^22,23^ have shown that improved noncovalent interactions can be obtained employing partially augmented (jun-cc-pV(n+d)Z) basis sets,^61,71^ and the corresponding model is labeled junChS. Replacement of conventional methods with the explicitly correlated (F12) variants leads to the junChSF12 model, which is even more accurate without any excessive additional cost. In detail, the starting point is the frozen-core (fc) CCSD(T)-F12b(3C/FIX) method^72−74^ again in conjunction with the jun-cc-pV(T+d)Z basis set.^61,75^ The corresponding auxiliary basis sets are also employed for resolution of the identity and density fitting, and the geminal exponent (γ) was fixed to 1.0 a_0_^–1^.^75,76^ CBS extrapolation is carried out with the standard *n*^–3^ two-point formula^77^ employing MP2F12/jun-cc-pV(X+d)Z energies with X = T and Q. The CV contribution is then incorporated as the difference between all-electron (ae) and fc MP2F12 calculations, both with the cc-pCVW(T+d)Z basis set.^78^ A systematic study of noncovalent intermolecular interactions^23^ showed that the junChSF12 approach is affected by small basis set superposition errors (BSSE), which would be difficult to take into account for intramolecular interactions. Furthermore, comparison with the most accurate results available for a panel of representative noncovalent complexes provided an average absolute error smaller than 10 cm^–1^.^22,23^

To determine the relative stability of different low-energy minima, one has to move from electronic energy differences to the corresponding relative enthalpies at 0K (*ΔH*_0_^°^) or free energies (Δ*G*°) at a temperature depending on the experimental conditions. The vibrational contributions to thermodynamic functions are usually computed by the harmonic oscillator (HO) model, which shows the largest errors in the high frequency (overestimated contributions to zero point energies) and low frequency (overestimated contributions to entropies) regions.

The first issue is solved in the present work by estimating anharmonic contributions in the framework of second order vibrational perturbation theory (VPT2), which provides analytical and resonance free expressions for the ZPEs.^79^ Harmonic (rDSD) and anharmonic (B3) contributions are employed in this connection, since a recent benchmark study has shown that for semirigid molecules the average absolute error of zero point energies with respect to accurate experimental results is reduced from 53 to 17 cm^–1^ when going from the HO to the VPT2 anharmonic model.^80^

The treatment of low frequency contributions (typically less than 100 cm^–1^) is more involved because different modes (e.g., torsions, inversions, etc.) need be identified, characterized, and treated by proper variational anharmonic computations.^36,81^ In the same benchmark study mentioned above in connection with anharmonic ZPEs,^80^ it has been shown that the simple one-dimensional hindered rotor model proposed by Ayala^82^ in conjunction with the VPT2 model for the other vibrational modes leads to remarkably accurate vibrational entropies for both semirigid and flexible molecules for which accurate experimental results are available. In particular, an average absolute error of 3 cm^–1^ is obtained for the *T*Δ*S* contribution to free energies at room temperature. In the present context, test computations showed that the unbiased detection of hindered rotations becomes ambiguous for some conformers, so that we prefer to resort to the much simpler and black-box quasi-harmonic (QH) approximation.^35,83^ In the QH approach, below a given cutoff value, entropic terms are obtained from the free-rotor model, and a damping function is used to interpolate between free-rotor and harmonic oscillator expressions close to the cutoff frequency.

The leading terms of MW spectra are the rotational constants of the vibrational ground-state (*B*_0_^*i*^, where *i* refers to the inertial axes *a*, *b*, *c*), which include vibrational corrections (Δ*B*_vib_^*i*^) in addition to equilibrium rotational constants (*B*_*e*_^*i*^).^84^ In the framework of the VPT2 approximation,^85^ the ground-state rotational constants can be expressed as
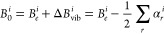
1where the α_*r*_’s are the vibration–rotation interaction constants and the sum runs over all *r* vibrational modes. Noted is that the evaluation of the α_*r*_’s implies anharmonic force field calculations and that the sum appearing in eq 1 (contrary to individual terms) does not involve any resonance issue at the VPT2 level (for details, see, e.g., refs (11, 86, 87)). Δ*B*_vib_^*i*^ being a small fraction of the corresponding *B*_*e*_^*i*^ (typically 0.5%),^88^ it can be determined at an affordable level of theory (B3 in the present context) without significantly affecting the accuracy of the resulting vibrational ground-state rotational constant.^11,89^ At the same time, inclusion of vibrational corrections is not warranted if the errors on the computed rotational constants are not much lower than 1% (50 MHz for a constant of 5000 MHz). Therefore, equilibrium rotational constants require very accurate geometrical parameters, which can be obtained only with state-of-the-art composite methods incorporating high excitation orders in the correlation treatment. These methods are able to deliver errors on equilibrium rotational constants as low as 0.1% (5 MHz for a rotational constant of 5000 MHz).^90^ The reduced cost junChSF12 composite method delivers typical relative errors of 0.2%,^11,80,91^ which are still sufficient for the unequivocal prediction and assignment of different conformers in the MW spectra of flexible molecules. Higher relative errors (typically 0.4–0.5%) are obtained at the rDSD level. However, the systematic nature of the errors permits geometrical parameters to be obtained and, thus, equilibrium rotational constants, rivaling the accuracy of the jun-ChSF12 counterparts by the linear regression approach (LRA). In this model, the computed geometrical parameters (*r*_*comp*_) are corrected for systematic errors by means of scaling factors (*a*) and offset values (*b*) depending on the nature of the involved atoms and determined once for ever from a large database of accurate semiexperimental (SE) equilibrium geometries:^38,92^
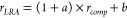
2The *a* and *b* values for different bonds and valence angles are taken from ref (38). Noted is that the intrinsic accuracy of the rDSD model leads in most case to *b* = 0.0 together with very small *a* values for bond lengths and that, among valence angles, only OCO and HCH need be corrected. Several studies have confirmed that very accurate molecular structures can be obtained employing this approach (referred to in the following as rDSD-LRA).^16,38,92,93^

Additional parameters of particular relevance for MW spectroscopy are the nuclear quadrupole coupling constants (χ_*ii*_, *i* referring to the inertia axis *a*, *b*, or *c*).^94^ Nuclear quadrupole coupling is the interaction between the quadrupole moment of a nucleus with nuclear spin *I* ≥ 1) and the electric gradient at the nucleus itself.^86^ Since at least one ^14^N quadrupolar nucleus is present in all amino acids, nuclear quadrupole coupling constants are important for accurate predictions of rotational spectra because they determine a splitting of the rotational transitions, which generates the so-called hypefine structure. Since a systematic study of rDSD quadrupole coupling constants has not yet been performed, the comparison with the experimental values for several conformers of different amino acids represents per se an interesting benchmark. We anticipate that vibrational effects on nuclear quadrupole coupling constants are usually smaller than the uncertainty affecting the computed equilibrium values, and thus they have not been considered in this work.

Finally, the components of dipole moments determine the intensities of rotational transitions and, as already mentioned, rDSD is expected to provide reliable values.^63^

Concerning technical details, the Gaussian package^95^ has been used for all calculations except the junChSF12 and QH ones, which have been performed with the help of the Molpro^76^ and GoodVibes^83^ software, respectively.

## 4 Structure and Soft Degrees of Freedom

**Figure 2:**
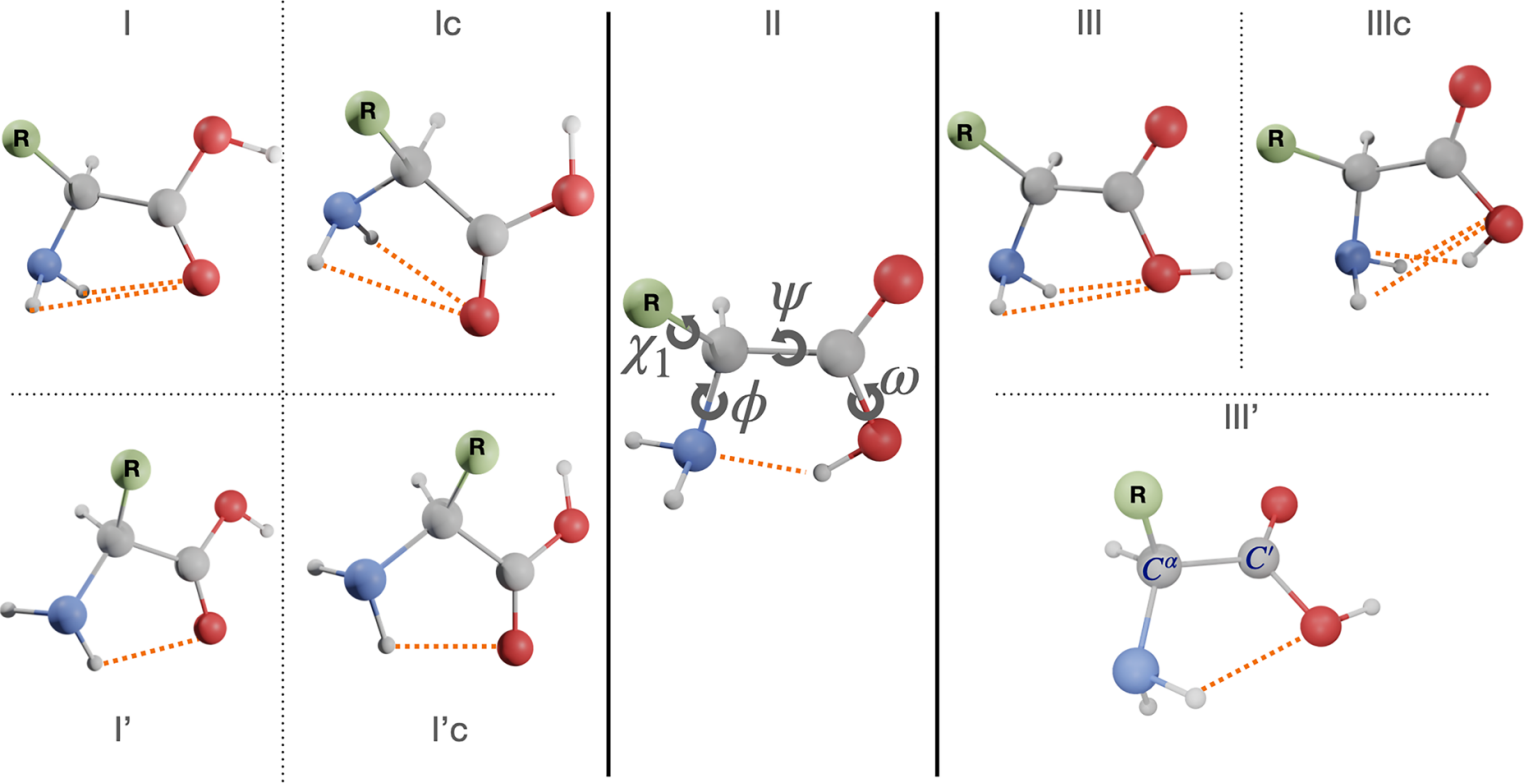
Structures of low-lying backbone conformers of α-amino acids.

The conformation of isolated amino acids is determined by both backbone (ϕ = H–N–C^α^–C′, ψ = N–C^α^–C′–O(H), and ω = C^α^–C′–O–H) and side chain (χ, defined more precisely in the following) torsional angles, as shown in the central panel of Figure 2. However, the nonplanarity of the NH_2_ moiety suggests replacing the customary ϕ dihedral angle (H–N–C^α^–C′) with ϕ′ = LP–N–C^α^–C′ = ϕ + 120°, where LP is the nitrogen lone-pair perpendicular to the plane defined by the two aminic hydrogens and the C^α^ atom.

The most stable backbone structures involve the formation of hydrogen bonds (see Figure 2), which can be classified as I (bifurcated NH_2_···O=C, ϕ′ ≈ 180°, ψ ≈ 180°, ω ≈ 180°), II (N···HO, ϕ′ ≈ 0°, ψ ≈ 0°, ω ≈ 0°), or III (bifurcated NH_2_···OH, ϕ′ ≈ 180°, ψ ≈ 0°, ω ≈ 180°).^4^ Higher energy minima can be classified as type I′ (single HNH···O=C hydrogen bond, ϕ′ ≈ 90°, ψ ≈ 180°, ω ≈ 180°) or type III′ (single HNH···OH hydrogen bond, ϕ′ ≈ 180°, ψ ≈ 90°, ω ≈ 180°). Furthermore, conformers of type I, I′, and III have higher energy counterparts for ω ≈ 0°, labeled in the following as Ic, I′c, and IIIc, respectively. The customary c, g, s, and t labels are used to indicate the cis, gauche, skew, and trans conformations for each dihedral angle in the order ϕ′,ψ, ω/χ_1_, ..., χ_*n*_.

For purposes of consistency with the original experimental studies, capital letters L, M, N, ... are used in some cases to label conformers of amino acids with polar side chains in order of decreasing relative populations estimated from MW spectra.^39,45,46,96^

## 5 Results and Discussion

### 5.1 The Smallest Prototypes: Glycine and Alanine

Glycine has been extensively characterized from both experimental and computational points of view (see refs (97−101) and references therein). Its limited size allowed the exploitation of state-of-the-art composite schemes including, together with CBS and CV contributions evaluated at the CCSD(T) level, also full account of triple excitations, perturbative inclusion of quadruple excitations, and relativistic contributions (CBS+CV+fT+pQ+rel).^101^ All the eight conformers mentioned above (I, II, III, I′, III′, Ic, IIIc, and I′c) have been characterized with four of them (I, III, Ic, and IIIc) having a planar backbone (*C*_*s*_ point group) and the other four (labeled with an asterisk to signal the presence of two equivalent nonplanar backbones) lacking any symmetry^98,99^ (see Table S2 of the SI). Concerning relative stabilities, the junChSF12 model performs remarkably well with an average absolute error of 6 cm^–1^ from the most accurate available results^101^ (see Table 1). The largest discrepancy (13 cm^–1^) is observed for the II conformer, which is slightly stabilized by triple and quadruple excitations. Also the accuracy of the rDSD model (maximum error (MAX) and MUE of 29 and 15 cm^–1^ with respect to the most accurate available results) is largely sufficient for most purposes and gives further support to the use of this computational level for geometry optimizations and harmonic frequency evaluations.

**Table 1:** Relative Electronic Energies (Δ*E*), Enthalpies at 0 K (Δ*H*_0_^°^ = Δ(E+ZPE)), and Free Energies at Room Temperature (Δ*G*°) (all in cm^–1^; 1 kJ/mol = 83.59 cm^–1^) for the Glycine Conformers

Conformer	Label	Δ*E*_*best*_^a^	Δ*E*_*ChS*_^b^	Δ*E*_*rDSD*_	Δ*H*_0H_^°^^c^	Δ*G*_*H*_^°^^d^	ΔZPE^e^	Δ(*T*Δ*S*)^f^	Δ*G*°^g^
ttt	I	0.0	0.0	0.0	0.0	0.0	0.0	0.0	0.0
ccc	II*	223.8	236.5	214.8	345.9	468.8	–38.5	–29.4	400.9
gtt	I′*	433.7	431.5	447.8	406.6	482.6	–23.2	–39.5	419.9
tct	III	605.1	605.8	583.5	630.1	239.6	6.7	215.3	461.6
gct	III′*	926.8	935.5	918.9	937.5	969.4	–9.2	1.8	962.0
ttc	Ic	1678.8	1688.7	1675.4	1616.5	1659.6	1.9	–20.4	1641.1
tcc	IIIc	2042.5	2051.5	2071.3	2131.9	2027.3	–10.8	–15.2	2001.3
gtc	I′c*	2119.4	2118.9	2140.6	2012.3	2085.7	–28.8	–35.6	2021.3

a CBS+CV+ft+fq+rel. from ref (101).

b JunChSF12 at rDSD geometries.

c JunChSF12 electronic energies with rDSD harmonic ZPE.

d JunChSF12 electronic energies with rDSD harmonic ZPE and thermal contributions.

e Difference between anharmonic and harmonic ZPEs at the B3 level.

f Difference between quasi-harmonic and harmonic *T*Δ*S* (see text for details).

g Sum of columns 7, 8, and 9.

Zero point and thermal contributions have a nonnegligible effect, leading to a significant destabilization of structure II and a strong stabilization of structure III (see Table 1). Inclusion of anharmonic contributions in ZPEs is needed for obtaining quantitative results but does not alter the stability order of the different conformers. Finally, the main effect of the QH corrections is to reduce the overstabilization of structure III produced by the harmonic oscillator model (see Table 1).

A shorter N···O distance in the II form with respect to I parallels the greater strength of the OH···N hydrogen bond with respect to its NH···O counterpart. Despite these relative hydrogen-bond strengths, the I conformer is more stable than II by about 230 cm^–1^ due to the more favorable (ω = 180° versus ω = 0°) arrangement of the carboxylic group in the I form. The role of the arrangement of the carboxylic group is confirmed by the nearly constant destabilization of the Ic and I′c forms with respect to their I and I′ counterparts (1690 cm^–1^ for Ic vs I and 1687 cm^–1^ for I′c vs I′). At the same time, the reduced stability of the III form with respect to I (about 600 cm^–1^) is related to the lower strength of the bifurcated NH_2_···O(H) hydrogen bond with respect to its NH_2_···O(=C) counterpart for identical arrangements of the carboxylic moiety. Finally conformers I′ and III′ are less stable than their I and III counterparts (by 430 and 330 cm^–1^, respectively) because a bifurcated hydrogen bond is replaced by a more conventional single hydrogen bond. This trend could change in the presence of polar side chains because it allows the formation of additional backbone (side chain) hydrogen bonds (vide infra).

Computation of energy barriers ruling the interconversion between pairs of adjacent conformers shows that structures III and I′ relax easily to structure I (with energy barriers of about 250 and 70 cm^–1^, respectively), whereas structure I′c relaxes to structure Ic (with an energy barrier of about 25 cm^–1^). Furthermore, the relative stability of structures III′ (927 cm^–1^), Ic (1679 cm^–1^), and IIIc (2043 cm^–1^) are too low to permit their unequivocal characterization by MW spectroscopy. We are thus left with only two conformers (I and II), which could be (and have actually been) detected in MW experiments.^40^

The availability of the experimental rotational constants for several isotopic species allowed the determination of very accurate semiexperimental equilibrium structures.^102^ For the I conformer, the MAX and MUE of rDSD geometrical parameters with respect to their semiexperimental counterparts are 0.0049 and 0.0019 Å for bond lengths and 0.46 and 0.15° for valence angles. The rDSD-LRA model does not change the situation for valence angles but reduces the errors of bond lengths by about five times (0.0008 and 0.0004), reaching the accuracy of state-of-the-art composite methods.^11,102^ More generally, all the computed spectroscopic parameters of the I and II conformers are in remarkable agreement with their experimental counterparts^40^ (see Table 2), with MAX and MUE of 30.2 and 13.6 MHz for rotational constants, 0.23 and 0.13 MHz for quadrupole coupling constants, and 0.1 and 0.05 D for dipole moment components. The errors for rotational constants and quadrupole coupling constants are close to those delivered by the ChS composite method (MAX and MUE of and 60.8 and 16.5 MHz for rotational constants 0.19 and 0.10 for quadrupole coupling constants).^99^ These results confirm that junChSF12 relative energies, rDSD-LRA structural parameters, and rDSD spectroscopic parameters can be confidently used for the comparison with experiments and represent reliable benchmarks for less refined quantum chemical methods.

**Table 2:** Rotational Constants (MHz), Quadrupole Coupling Constants (χ in MHz), and Dipole Moment Components (μ in debye) of the Detected Conformers of Glycine

Parameter	Iexp^a^	Icalc^b^	IIexp^a^	II calc^b^
*A*_0_	10341.5279(49)	10311.35	10130.1521(57)	10144.00
*B*_0_	3876.1806(23)	3865.70	4071.5120(17)	4059.68
*C*_0_	2912.3518(16)	2904.74	3007.4852(14)	2999.51
χ_*aa*_	–1.208(9)	–1.336	1.773(2)	1.922
χ_*bb*_	–0.343(8)	–0.448	–3.194(4)	–3.344
χ_*cc*_	1.551(9)	1.785	1.421(4)	1.422
μ_*a*_	0.911(3)	1.01	5.372(34)	5.39
μ_*b*_	0.607(5)	0.66	0.93(1)	0.83
μ_*c*_	0.0	0.0	0.0	0.03

a From ref (40). Standard errors are given in parenthesis in units of the last digit.

b rDSD-LRA equilibrium geometries, rDSD equilibrium properties, and B3 vibrational corrections (only for rotational constants).

Moving to alanine,^41,103−108^ the two sides of the average backbone plane are no longer equivalent, with two nearly iso-energetic minima (corresponding to positive or negative values of the ψ dihedral angle) being expected at least for structures of II, I′, III′, and I′c type. The number of conformers thus increases to 12, but unconstrained geometry optimizations lead also to a splitting of structure III into III and III^–^, although the energy difference is so tiny that an effective planar structure is expected. In all the energy minima the methyl group is found in a staggered position with respect to the substituents at C^α^ with rotational barriers of about 1200 cm^–1^, close to the value of 1140 cm^–1^ obtained for ethane at a comparable computational level.^109^

The MAX and MUE of rDSD computations with respect to the junChSF12 reference (42.1 and 15.7 cm^–1^) are more than five times smaller than the corresponding B3 values (222.2 and 91.6 cm^–1^) and less than half the corresponding MP2 values (96.5 and 28.8 cm^–1^). What is even more important, junChSF12 and rDSD provide the same stability order, whereas B3 and MP2 computations overestimate the stability of type II conformers (see Table S3 of the SI).

As already mentioned, the comparison with experiment requires the computation of the relative free energies for the different conformers at the temperature of the carrier gas (in order to evaluate their population) and of transition states ruling their interconversion. The results collected in Table 3 show that all the conformers involving ω values around 0° (Ic, III^–^c, I′^–^c, and I′c) are too unstable to permit their unequivocal detection in MW experiments. Furthermore, relaxation of I′ and III′ conformers to their more stable I and III counterparts is ruled by low energy barriers, which are easily overcome in the typical conditions of supersonic-jet expansion. Low energy barriers govern also the relaxation of III to I and II to II^–^ conformers. As a consequence, only the I and II^–^ conformers could be detected in MW studies, with the former collecting the populations of I, III^–^, III, I′, I′^–^, III′, and III′^–^ conformers and the latter those of the II and II^–^ conformers. It is remarkable that the relative population of conformer I computed at room temperature from the free energies collected in Table 1 (76%) is in good agreement with the experimental estimate (80%),^41^ whereas a significantly lower relative population (54%) would have been predicted neglecting zero point and thermal effects.

**Table 3:** Relative Electronic Energies (Δ*E*), Enthalpies at 0 K (Δ*H*_0_^°^ = Δ(E+ZPE)), and Free Energies at Room Temperature (Δ*G*°) for the Alanine Conformers^a^

Conformer	Label	Δ*E*_*ChS*_^b^	Δ*E*_*rDSD*_	Δ*H*_0H_^°^^c^	Δ*G*_*H*_^°^^d^	ΔZPE^e^	Δ(*T*Δ*S*)^f^	Δ*G*°^g^
ttt	I	0.0	0.0	0.0	0.0	0.0	0.0	0.0
cg^–^c	II^–^	35.6	29.5	172.5	316.5	–28.1	–46.8	241.6
cgc	II	103.1	106.4	215.6	321.4	–26.7	–20.4	274.3
tg^–^t	III^–^	432.6	412.8	443.0	386.4	–12.5	42.1	416.0
tgt	III	436.0	410.1	452.1	329.9	h	–81.0	h
ga^–^t	I′	396.5	406.7	389.7	448.0	6.5	–25.7	428.8
gat	I′^–^	446.6	480.5	425.9	490.4	–8.1	–19.8	462.5
ggt	III′	613.5	655.6	592.4	631.7	4.0	–0.8	634.9
gg^–^t	III′^–^	789.7	782.7	774.3	804.6	1.3	–5.7	800.2
tsc	Ic	1736.0	1730.3	1681.6	1696.1	–0.7	–16.0	1679.4
ts^–^c	III^–^c	1980.5	1968.5	1928.2	2006.6	–54.3	–12.5	1839.8
g^–^tc	I′c	2116.5	2123.5	2052.8	2105.8	–28.1	–24.8	2052.9
gtc	I′^–^c	2154.9	2165.3	2043.0	1983.5	27.3	25.2	2036.0

a All the data are in cm^–1^.

b JunChSF12 at rDSD geometries.

c JunChSF12 electronic energies with rDSD harmonic ZPE.

d JunChSF12 electronic energies with rDSD harmonic ZPE and thermal contributions.

e Difference between anharmonic and harmonic ZPE at the B3 level.

f Difference between quasi-harmonic and harmonic *T*Δ*S* (see text for details).

g Sum of columns 6, 7, and 8.

h No minimum at the B3 level.

Table 4 collects the experimental and computed rotational parameters for the I and II^–^ conformers. A remarkable agreement is noted with the MAX and MUE of rDSD-LRA/B3 rotational constants (36.1 and 10.5 MHz) being even better than those (50.5 and 12.2 MHz) obtained at the much more expensive CCSD(T)/cc-pVTZ level.^106^ It is noteworthy that for both conformers of alanine the error on the *B*_0_ rotational constant is much higher than those affecting the other two rotational constants, whereas in both the observed conformers of glycine the largest error was found for *A*_0_.

**Table 4:** Rotational Constants and Quadrupole Coupling Constants (χ) in MHz of the Detected Conformers of Alanine

Parameter	Iexp^a^	Icalc^b^	IIAexp^a^	IIAcalc^b^
*A*_0_	5066.1455(7)	5061.61	4973.0546(35)	4972.03
*B*_0_	3100.9507(5)	3070.85	3228.3375(56)	3192.26
*C*_0_	2264.0131(4)	2273.39	2307.8090(42)	2326.00
χ_*aa*_	–3.2567(11)	–3.4864	0.4515(17)	0.8298
χ_*bb*_	2.0093(16)	1.9918	0.3267(21)	0.4207
χ_*cc*_	1.2474(16)	1.4946	–0.7782(21)	–1.2505

a From ref (41). Standard errors are given in parenthesis in units of the last digit.

b rDSD-LRA equilibrium geometries, rDSD properties, and B3 vibrational corrections.

The geometrical parameter most sensitive to conformational changes is the NC^α^C′ valence angle, which decreases by about 3.5° when going from the I to the II^–^ conformer, consistent with the trans-angle rule of hyperconjugative and steric effects.^110^ At the same time, the C=O bond length shows the expected lengthening by about 0.002–0.003 Å when going from free (structure II^–^) to hydrogen-bonded (structure I) forms.

The only significant differences between the geometrical parameters of glycine and those of alanine concerns the C^α^–C′ bond length (shorter in glycine by about 0.007 Å for both conformers) and the NC^α^C′ valence angle (narrower in glycine by about 2° for both conformers). Therefore, the main structural differences between glycine and alanine are highly localized at the C^α^. As already mentioned, the ψ torsional angle characterizes the backbone deviation from planarity (see Tables S2 and S3 of the SI). For I conformers, it is exactly equal to 180° in glycine, whereas the lack of any symmetry induces a change of more than 15° in alanine. On the other hand, comparable ψ values are observed for the II forms of glycine and alanine (12° and 15°, respectively).

### 5.2 Amino Acids with Polar Side Chains

Systematic investigations have revealed that, in analogy with alanine, the natural amino acids containing simple nonpolar side chains (valine,^42^ isoleucine,^43^ and leucine^44^) present two dominant conformers of types I and II, respectively. On the other hand, the conformational landscape of natural amino acids with polar side chains is much richer due to the synergy or competition between intrabackbone and backbone (side chain) hydrogen bonds.

Let us start our discussion from serine (Ser), which has two soft degrees of freedom in its CH_2_OH side chain (χ_1_ = N–C^α^–C^β^–O and χ_2_ = C^α^–C^β^–O–H), with the OH moiety able to act either as donor or acceptor in quite strong intramolecular hydrogen bonds.^111^ The increased number of soft degrees of freedom (from 3 to 5) makes this system suitable for applying the PES exploration strategy introduced in the previous sections, which produces 12 low-energy conformers (see Table 5).

**Table 5:** rDSD Relative Electronic Energies, Harmonic Zero Point Energies, Thermal Contributions, and Quasi-harmonic Corrections, together with Difference with JunChSF12 Electronic Energies and B3 Anharmonic Corrections (all in cm^–1^) for the Low-Lying Conformers of Serine^a^

Label	Δ*E*_*rDSD*_	ΔChS	ΔZPE_*H*_	ΔTh_*H*_	ΔZPE_(*anh*–*H*)_	*T*Δ*S*_(*QH*–*H*)_	Δ*G*°^b^	ϕ′	ψ	ω	χ_1_	χ_2_
IIgg	0.0	0.0	0.0	0.0	0.0	0.0	0.0	–33.7	21.9	–6.2	59.4	79.3
Ig^–^g	161.8	11.2	–121.8	–113.1	21.3	7.7	–32.9	159.3	166.5	177.3	–55.9	44.1
IItg^–^	222.2	11.4	24.3	54.7	–10.0	–39.7	262.9	–31.5	19.2	–4.5	–171.9	–54.2
I′gg^–^	337.7	–43.1	–122.4	–34.9	9.3	–11.4	135.2	95.1	–173.4	–180.0	57.1	–46.9
III′gg	531.9	–0.4	–60.7	–54.6	10.9	9.6	436.7	–168.9	67.2	–177.1	59.4	68.6
IIg^–^t	602.4	32.2	–71.9	–28.3	15.9	–39.5	510.8	30.3	–14.2	2.4	–60.0	178.3
III′tg^–^	792.7	8.2	–32.6	–192.3	–2.7	11.4	584.7	178.0	64.9	–178.3	–178.4	–70.6
IIg^–^g^–^c	607.9	43.0	–46.5	–9.6	–12.6	–11.0	571.2	29.8	–15.7	3.4	–58.6	–76.9
III′g^–^g^d^	731.9	34.1	–120.7	–197.7	5.3	61.9	514.8	165.8	–27.4	–176.7	–56.4	43.0
IIg^–^t^e^	759.4	35.0	–129.3	–81.6	5.0	–14.0	574.5	–34.9	18.5	–3.6	–58.9	–174.3
Igt^f^	853.1	4.5	–225.8	–131.5	6.5	5.3	512.1	–169.6	–179.6	–179.1	65.1	–175.6
Igg^g^	869.3	–6.9	–188.3	–185.0	9.6	11.4	510.1	–164.3	–165.8	–176.5	66.4	83.3

a Best estimates of relative free energies at room temperature (Δ*G*° in cm^–1^) and dihedral angles optimized at the rDSD level (ϕ′, ψ, ω, χ_1_ = N–C^α^–C^β^–O and χ_2_ = −C^α^–C^β^–O–H in degrees) are also given. See main text for details.

b Sum of columns 2, 3, 4, 5, 6, and 7.

c Relaxes to IIg^–^t.

d Relaxes to Ig^–^g.

e Relaxes to the other IIg^–^t form.

f Relaxes to Igg^–^.

g Relaxes to III′gg.

**Figure 3:**
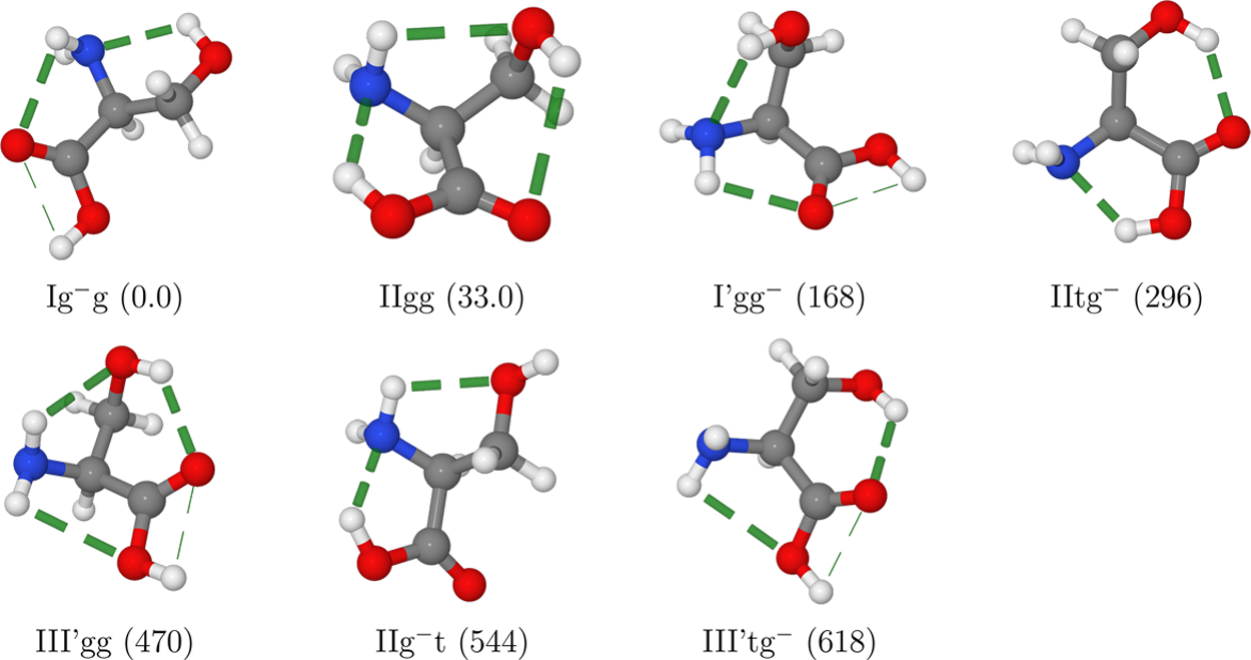
Representations of the seven serine conformers detected in MW spectra with the computed relative free energies at room temperature (in cm^–1^) given in parentheses. H-bonds are highlighted by dashed lines.

However, the IIg^–^g^–^ conformer relaxes to the more stable IIg^–^t form through rotation around χ_2_; IIIg^–^g relaxes to Ig^–^g through rotation around ψ; the less stable IIg^–^t conformer relaxes to its more stable counterpart through a planar structure (invert ϕ′, ψ, and ω); Igt relaxes to I′gg^–^ through rotation around χ_2_, and Igg relaxes to III′gg through rotation around ψ. We are thus left with seven conformers possibly detectable in MW experiments: three of type II, two of type III′, and one each for types I and I′ (see Figure 3).

All the most stable conformers are stabilized by both intrabackbone and backbone (side chain) hydrogen bonds (see Figure 3). Furthermore, contrary to III conformers, III′ structures are locked in sufficiently deep wells to become detectable by one HNH···OH (III′gg) or OH···O=C (III′tg^–^) hydrogen bond between the backbone and the side chain in addition to the intrabackbone HNH···OH hydrogen bond.

ZPEs and thermal contributions alter the ordering of the four most stable conformers stabilizing, as usual, structures of type I with respect to their type II counterparts. The stability order provided by the computed free energies at room temperature matches perfectly the estimate based on the relative intensities of the MW signals.^45^ According to both theory and experiments, the first four conformers (one of type I, one of type I′, and two of type II) are significantly more stable than the two conformers of type III′ and a further conformer of type II, which have, in turn, comparable stability (see Table 6).

**Table 6:** Ground-State Rotational Constants (*A*_0_, *B*_0_, and *C*_0_ in MHz), ^14^N-Nuclear Quadrupole Coupling Constants (χ in MHz), and Electric Dipole Moment Components (μ in debye) of the Seven Most Stable Serine Conformers^a^

Calc.^b^	Ig^–^g	IIgg	I′gg^–^	IItg^–^	III′gg	IIg^–^t	III′tg^–^
*A*_0_^*c*^	4461.34	3549.33	3505.74	3630.86	3950.32	4508.13	3464.84
*B*_0_^*c*^	1823.01	2372.38	2305.21	2382.52	2222.91	1843.00	2304.68
*C*_0_^*c*^	1441.95	1734.67	1803.62	1515.28	1657.03	1462.05	1604.74
χ_*aa*_	–4.5535	–3.6696	–0.9235	–3.8114	–0.6094	–0.3660	–1.0975
χ_*bb*_	2.8681	2.1341	2.5528	2.1268	–0.6702	2.0569	–0.6582
χ_*cc*_	1.6854	1.5355	–1.6293	1.6847	1.2796	–1.6909	1.7557
μ_*a*_	1.8574	2.1328	–0.4050	–0.7709	–2.5568	4.0962	–2.8253
μ_*b*_	–0.2255	–3.1566	–0.7361	4.8433	–0.2893	–1.7795	–0.5939
μ_*c*_	0.7853	–1.4660	–2.7540	–0.1467	–0.5279	0.2594	0.5548
Δ*G*^0^	0.0	32.9	168.1	295.8	469.2	543.7	617.6

Exp.^c^	L	M	N	O	P	R	Q
*A*_0_	4479.0320(12)	3557.20088(35)	3524.38806(41)	3638.05784(38)	3931.7548(76)	4517.473(17)	3510.4015(35)
*B*_0_	830.16170(25)	2380.37208(40)	2307.76826(70)	2387.89651(99)	2242.76701(70)	1846.99360(30)	2321.90829(24)
*C*_0_	1443.79545(28)	1740.92458(10)	1805.20788(60)	1519.18716(36)	1664.53012(57)	1463.79646(31)	1584.38608(32)
χ_*aa*_	–4.3023(27)	–3.4616(19)	–1.1343(35)	–3.6257(57)	–0.6733(67)	–0.6066(55)	–1.0486(55)
χ_*bb*_	2.82359(63)	2.07974(93)	2.5043(50)	2.06213(26)	–0.456(16)	2.0723(82)	–0.5637(53)
χ_*cc*_	1.4788(46)	1.3819(47)	–1.3701(50)	1 0.5906(50)	1.129(16)	–1.466(30)	1.612(21)

a Relative free energies at room temperature (Δ*G*^0^ in cm^–1^) are also reported.

b Computed data are at the rDSD level (including LRA corrections for equilibrium rotational constants) except for electronic energies (junChSF12) and vibrational corrections to equilibrium rotational constants (B3).

c Standard errors are shown in parentheses in units of the last digits.

The rotational constants of the two most stable conformers have been recently computed by geometry optimizations at the ChS level, reaching MAX and MUE of 28.7 and 10.6 MHz, respectively.^23,112^ It is noteworthy that even smaller MAX and MUE (17.7 and 8.1 MHz, respectively) are obtained at the rDSD-LRA level, whose strongly reduced cost has allowed us to compute the spectroscopic parameters of all the other low-energy conformers. The remarkable agreement between computed and experimental results for all the detected conformers of serine confirms the accuracy of our computational strategy.

The next studied system is threonine (Thr),^113^ in which a methyl group replaces one of the hydrogen atoms bonded to C^β^, leading to the CHCH_3_OH side chain which has again two soft degrees of freedom (χ_1_ = N–C^α^–C^β^–O and χ_2_ = C^α^–C^β^–O–H) since the terminal methyl group is frozen in a staggered conformation with an estimated rotation barrier of 1400 cm^–1^. There is now a second chiral center in addition to the C^α^ atom, with the natural amino acid being 2*S*,3*R*-threonine. The conformational landscape of threonine has been investigated in two different studies,^113,114^ which obtained 71 and 56 conformers, respectively, in a range of about 4000 cm^–1^, but the final set of conformers was the same up to a relative energy of 1600 cm^–1^. The knowledge-based step of our conformational exploration started from the 12 low-energy conformers of serine collected in Table 5, each of them being then split into two nonequivalent structures. Next, the IM-EA algorithm was used to generate additional low-energy minima. At the end of these two steps and the subsequent filtering/refinement we are left with the 10 low-energy conformers (within an energy range of 1000 cm^–1^) collected in Table 7. It is noteworthy that this finding is in full agreement with ref (114).

**Table 7:** rDSD Relative Electronic Energies, Harmonic Zero Point Energies, Thermal Contributions, and Quasi-harmonic Corrections, together with Difference with JunChSF12 Electronic Energies and B3 Anharmonic Corrections (all in cm^–1^) for the Low-Lying Conformers of Threonine^a^

Label	Δ*E*_*rDSD*_	ΔChS	ΔZPE_*H*_	ΔTh_*H*_	ΔZPE_(*anh*–*H*)_	*T*Δ*S*_(*QH*–*H*)_	Δ*G*°^b^	ϕ′	ψ	ω	χ_1_	χ_2_
IIgg	0.0	0.0	0.0	0.0	0.0	0.0	0.0	–33.7	21.8	–6.1	60.0	77.1
Ig^–^g	218.5	34.5	–85.9	–83.0	27.7	20.4	132.2	162.5	143.7	177.8	–55.4	42.4
IItg^–^	371.5	2.8	24.4	58.6	20.1	–30.5	446.9	–25.4	13.1	–2.3	–168.8	–53.7
I′gg^–^	459.8	–36.4	–93.7	13.4	21.3	–14.7	349.7	99.6	–175.8	–179.2	56.9	–47.3
III′g^–^g	574.6	45.8	–119.6	–135.4	44.4	45.4	455.2	168.2	–51.1	–179.4	–56.1	42.1
III′gg	624.2	10.8	–76.8	–62.6	–37.4	4.4	462.6	–170.6	72.6	–176.4	57.8	65.8
IIg^–^t	711.2	9.2	–68.8	–25.5	7.5	–8.1	625.5	35.1	–21.0	5.4	–54.6	–177.8
IIgt^c^	586.1	6.9	–137.1	–104.9	–48.5	9.5	312.0	–26.2	11.5	–2.6	50.1	161.2
IIg^–^g^–^	725.9	24.4	–5.8	–9.0	0.1	–7.5	728.1	34.1	–21.8	6.1	–51.8	–84.1
Igt^d^	962.3	8.8	–242.2	–137.4	4.0	8.4	603.9	–172.7	178.6	–179.4	64.0	179.4

a Best estimates of relative free energies at room temperature (Δ*G*° in cm^–1^) and dihedral angles optimized at the rDSD level (ϕ′, ψ, ω, χ_1_ = N–C^α^–C^β^–O, and χ_2_ = C^α^–C^β^–O–H in degrees) are also given. See main text for details.

b Sum of columns 2, 3, 4, 5, 6, and 7.

c Relaxes to IIgg.

d Relaxes to Ig^–^g.

**Figure 4:**
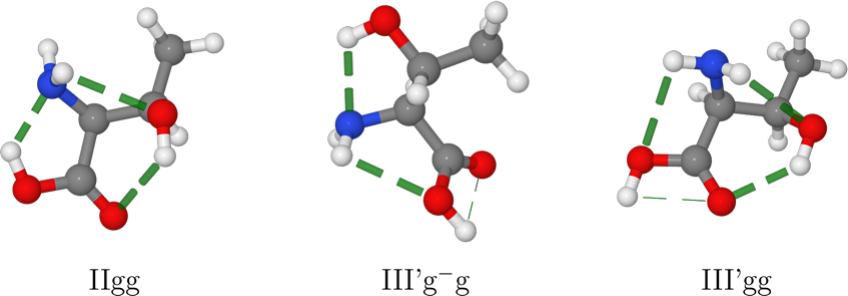
Absolute energy minimum and low-lying III′ conformers of threonine. The H-bonds are highlighted by dashed lines.

**Figure 5:**
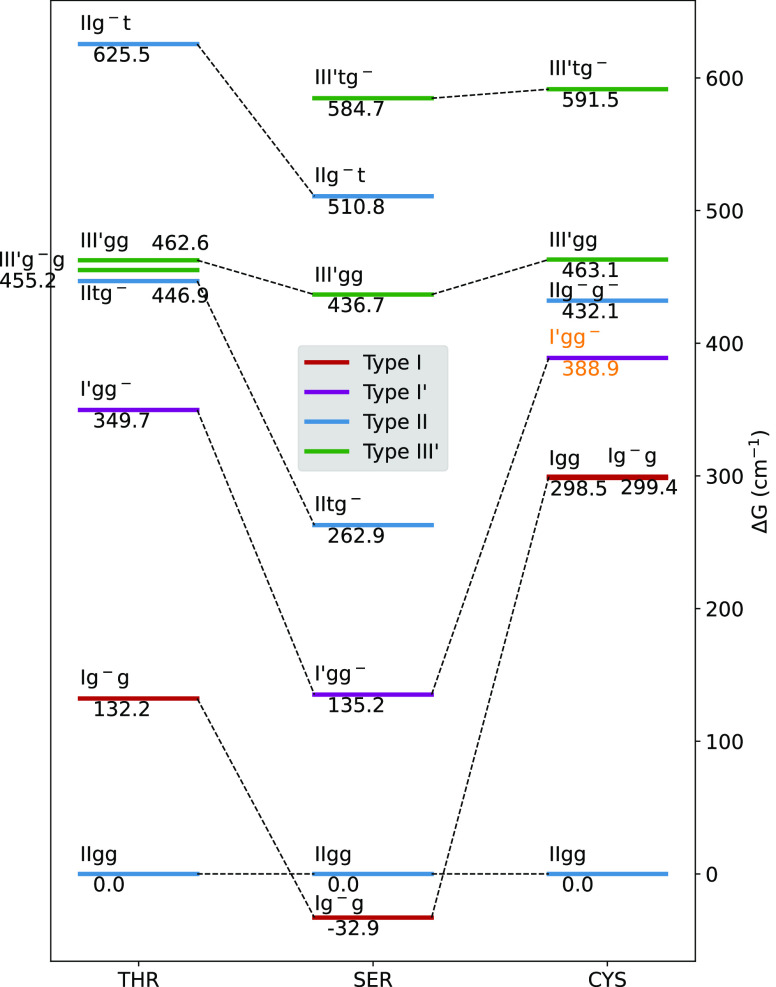
Observed conformers of threonine, serine, and cysteine. The relative free energies at room temperature (Δ*G* in cm^–1^, see text for details) are given for each amino acid with respect to its IIgg conformer. The relations between the observed conformers of the three amino acids are highlighted with dashed lines. The conformer I′gg^–^ of cysteine, which has not been detected in MW studies, is reported with orange labels.

The predicted population of conformer IIg^–^g^–^ is too low to allow its detection in MW experiments, and conformers IIgt and Igt relax easily to conformers IIgg and Ig^–^g, respectively. We are thus left with the same number (seven) and backbone conformation (three conformers of type II, two of type III′, and one each for types I and I′) of the structures discussed above for serine, which should be (and have actually been^46^) detected in MW experiments. However, the presence of the β methyl group increases the energy barrier governing relaxation of the III′g^–^g conformer to its Ig^–^g counterpart from about 200 to about 800 cm^–1^ when going from serine to threonine. As a consequence, the III′g^–^g conformer is observed in threonine in place of the less stable III′tg^–^ conformer observed in serine (see Figure 4). At the same time, a general destabilization of all conformers with respect to IIgg accompanies the substitution of a β hydrogen atom with a methyl group (see Figure 5).

The two most stable (IIgg and Ig^–^g) and the three least stable (III′g^–^g, III′gg, and IIg^–^t) conformers are the same in terms of electronic energies, enthalpies, or free energies. The relative ordering of the two intermediate conformers is, instead, altered by both ZPE and thermal contributions.

All the spectroscopic parameters of the seven low-energy conformers of threonine detected in a recent microwave study^46^ show a remarkable agreement with those computed for the most stable conformers predicted by our computations (see Table 8). The relative stability order estimated from the experimental results is Ig^–^g > IIgg > I′gg^–^ > IIg^–^t ≈ III′g^–^g ≈ IItg^–^ ≈ III′gg, which is in general agreement with the computed relative free energies except for the inversion between Ig^–^g and IIgg conformers and the position of the IIg^–^t structure.

**Table 8:** Ground-State Rotational Constants (*A*_0_, *B*_0_, and *C*_0_ in MHz), ^14^N-Nuclear Quadrupole Coupling Constants (χ in MHz), and Electric Dipole Moment Components (μ in debye) of the Seven Most Stable Conformers of Threonine^a^

	IIgg	Ig^–^g	I′gg^–^	IItg^–^	III′g^–^g	III′gg	IIg^–^t
Computed^b^							
*A*_0_	3223.67	2864.48	3141.58	2671.88	2885.67	3375.76	2907.62
*B*_0_	1528.34	1602.22	1501.39	1774.76	1564.86	1474.72	1656.40
*C*_0_	1265.11	1214.77	1313.14	1376.76	1243.85	1234.69	1187.62
χ_*aa*_	–3.5846	–4.3527	–0.2035	–4.1467	–4.2988	–2.2144	–0.3702
χ_*bb*_	1.7308	2.6918	2.9006	2.4728	2.5948	–0.2748	2.6214
χ_*cc*_	1.8538	1.6609	–2.6971	1.6739	1.7040	2.4892	–2.2513
μ_*a*_	2.85	–2.06	–0.19	0.04	–1.86	–2.23	3.78
μ_*b*_	2.95	0.01	–0.34	4.91	1.58	0.98	2.00
μ_*c*_	–0.94	0.97	–2.90	–0.16	1.33	–0.87	0.19
Δ*G*^0^	0.0	132.2	349.7	446.9	455.2	462.6	625.5
Experimental^c^							
*A*_0_	3232.4827(12)	2872.77049(48)	3148.59247(32)	2670.72096(53)	2889.93352(45)	3379.841(14)	2912.6227(20)
*B*_0_	1533.71801(32)	1608.95699(26)	1506.27679(37)	1784.66894(60)	1572.32152(50)	1482.04984(21)	1660.21807(34)
*C*_0_	1267.88615(34)	1211.39762(38)	1316.33575(44)	1383.75384(51)	1241.83423(47)	1237.59121(22)	1189.31443(34)
χ_*aa*_	–3.4971(21)	–4.1859(25)	–0.7403(21)	–3.7652(73)	–4.1529(32)	–2.201(14)	–0.544(11)
χ_*bb*_	1.7519(27)	2.661(42)	2.8781(28)	2.4258(75)	2.5682(46)	–0.157(50)	2.582(16)
χ_*cc*_	1.7452(60)	1.5248(17)	–2.1378(70)	1.3394(20)	1.5846(46)	2.358(64)	–2.038(50)

a The computed relative free energies at room temperature (Δ*G*^0^ in cm^–1^) are also reported.

b Computed data are at the rDSD level (including LRA corrections for equilibrium rotational constants) except for electronic energies (junChSF12) and vibrational corrections to equilibrium rotational constants (B3).

c Standard errors are shown in parentheses in units of the last digits.

Replacement of the oxygen atom in the side chain of serine by a sulfur produces cysteine (Cys), whose CH_2_SH side chain has again two soft degrees of freedom (χ_1_ = N–C^α^–C^β^–S and χ_2_ = C^α^–C^β^–S–H). One might think that the same conformers should be detected for cysteine and serine. However, the strengths of the H-bonds possibly formed by the thiol group are weaker than those of its alcohol counterpart. Therefore, it is expected that the barriers separating low-lying conformers decrease and in some instances may even disappear. In ref (115), a systematic scan of the conformational PES at the MP2/cc-pVTZ level led to the identification of 71 unique conformers, thus defining a reference data set. The knowledge-based step of our PES exploration involved the 12 low-energy conformers found for serine and integration of these structures with those issued from the IM-EA exploration employing sufficiently high energy thresholds allowed us to retrieve all the structures of the reference data set.^115^ Then, refinement of the results by the usual energy tresholds led to the 9 conformers collected in Table 9. The rDSD results are once again in very good agreement with their junChSF12 counterparts (MAX and MUE of 44 and 24 cm^–1^, respectively).

**Table 9:** rDSD Relative Electronic Energies, Harmonic Zero Point Energies, Thermal Contributions, and Quasi-harmonic Corrections, together with Difference with JunChSF12 Electronic Energies and B3 Anharmonic Corrections (all in cm^–1^) for the Low-Lying Conformers of Cysteine^a^

Label	Δ*E*_*rDSD*_	ΔChS	ΔZPE_*H*_	ΔTh_*H*_	ΔZPE_(*anh*–*H*)_	*T*Δ*S*_(*QH*–*H*)_	Δ*G*°^b^	ϕ′	ψ	ω	χ_1_	χ_2_
IIgg	0.0	0.0	0.0	0.0	0.0	0.0	0.0	–32.7	18.6	–4.8	57.1	71.8
IIg^–^g^–^	501.1	36.3	–24.6	–53.3	–7.6	–19.8	432.1	34.4	–18.0	4.0	–60.9	–65.4
Igg	571.1	–9.3	–177.6	–196.9	47.8	63.4	298.5	–171.3	–175.8	–177.4	63.7	74.7
Ig^–^g	630.7	8.9	–180.6	–198.4	10.0	28.8	299.4	162.9	162.6	177.5	–65.2	51.0
III′gg	706.7	–18.5	–123.9	–168.2	1.6	65.4	463.1	–172.3	34.5	177.4	61.6	76.1
III′tg^–^	873.8	39.0	–153.0	–185.5	–22.5	39.7	591.5	175.6	85.6	–175.8	–175.4	–75.8
I′gg^–^c	722.3	–44.1	–201.0	–85.4	1.2	–4.2	388.8	98.9	–173.0	179.8	64.3	–52.5
III′gg^–^	950.0	41.2	–79.1	–164.4	–15.2	19.3	751.8	114.0	79.9	–65.7	88.3	16.7
IIgt	1056.5	–0.4	–38.8	–24.8	17.0	–13.8	995.7	152.9	1.8	–27.2	100.7	27.0

a Best estimates of relative free energies at room temperature (Δ*G*° in cm^–1^) and dihedral angles optimized at the rDSD level (ϕ′, ψ, ω, χ_1_ = N–C^α^–C^β^–S, and χ_2_ = C^α^–C^β^–S–H in degrees) are also given. See main text for details.

b Sum of columns 2, 3, 4, 5, 6, and 7.

c Relaxes to Ig^–^g.

**Figure 6:**
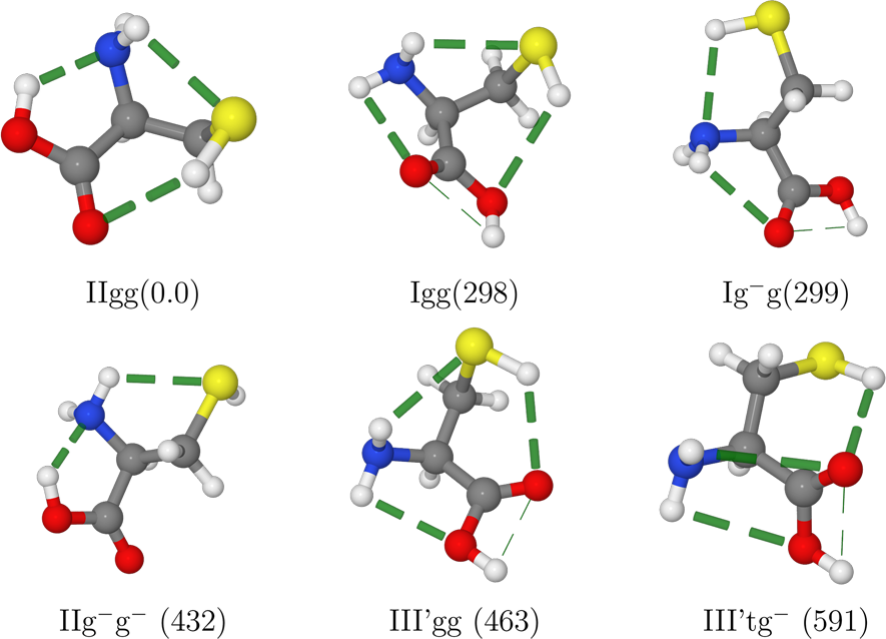
Cysteine conformers detected in MW experiments with the computed relative free energies at room temperature (in cm^–1^) given in parentheses. H-bonds are highlighted by dashed lines.

Among those nine conformers, the two least stable ones have too low populations to allow their unequivocal experimental characterization and the conformer I′gg^–^ relaxes easily to its Ig^–^g counterpart, which has a similar shape. Therefore, the number of detectable conformers reduces to 6: two each for types I, II, and III′ (see Figure 6). The backbone structure of the most stable conformer and the general trends are similar to those discussed above for serine and threonine (see Figure 5), but the conformers Igg and IIg^–^g^–^ replace the I′gg^–^ and IIg^–^t counterparts observed in both serine and threonine.

The spectroscopic parameters computed at the rDSD level are in remarkable agreement with their experimental counterparts^39^ with MUEs of 11.7, 5.7, and 3.1 MHz for the *A*_0_, *B*_0_, and *C*_0_ rotational constants, respectively (Table 10). The errors on *B*_0_ and *C*_0_ are quite low already at the rDSD level (see Table S9 of the SI), whereas errors as large as 40 MHz are obtained for the *A*_0_ rotational constant. For most conformers, the C–S bond is nearly perpendicular to the average backbone direction (see Figure 6) and is, in turn, roughly aligned with the *a* axis. As a consequence, any overestimation of the C–S bond length results in a nonnegligible underestimation of the *A*_0_ rotational constant. In this connection, the LRA correction brings the computed values in remarkable agreement with experiment (the maximum error is obtained for the Ig^–^g conformer and amounts to 18 MHz, i.e., 0.4%).

**Table 10:** Ground-State Rotational Constants (*A*_0_, *B*_0_, and *C*_0_ in MHz), ^14^N-Nuclear Quadrupole Coupling Constants (χ in MHz), and Electric Dipole Moment Components (μ in debye) of the Six Most Stable Energy Minima of Cysteine^a^

Calc.^b^	IIgg	Igg	Ig^–^g	III′gg	IIg^–^g^–^	III′tg^–^
*A*_0_	3063.27	2874.44	4217.57	3223.13	4352.34	2989.53
*B*_0_	1600.59	1615.60	1181.79	1563.71	1173.71	1524.30
*C*_0_	1327.34	1366.95	1000.82	1267.50	1012.74	1210.12
χ_*aa*_	–3.3302	–0.0280	–4.5456	0.0509	–0.1942	0.5818
χ_*bb*_	2.5198	0.3553	2.8019	–0.5218	2.2497	–2.1507
χ_*cc*_	0.8104	–0.3273	1.7437	0.4708	–2.0555	1.5689
μ_*a*_	1.40	–1.02	–1.81	2.86	2.33	–2.12
μ_*b*_	3.98	–1.43	0.37	–2.42	–0.18	0.31
μ_*c*_	–1.53	–1.39	0.57	1.36	–0.20	–0.02
Δ*G*^0^	0.0	187.3	260.6	396.1	459.5	574.3

Exp.^c^	O	N	L	P	M	Q
*A*_0_	3071.1437(15)	2889.44652(93)	4235.63210(58)	3216.218(26)	4359.22320(77)	3004.1689(90)
*B*_0_	1606.53664(36)	1622.99829(32)	1187.27897(20)	1572.74943(63)	1178.27610(13)	1527.40718(53)
*C*_0_	1331.80185(34)	1367.83448(26)	1003.10663(23)	1276.79135(55)	1015.27433(13)	1210.70722(46)
χ_*aa*_	–3.1200(53)	–0.1465(36)	–4.263(11)	0.0	–0.4060(9)	0.505(10)
χ_*bb*_	2.4418(61)	0.4419(43)	2.776(11)	–0.449(25)	2.2314(43)	–1.991(20)
χ_*cc*_	0.6782(61)	–0.2954(43)	1.488(11)	0.449(25)	–1.8254(43)	1.486(20)

a The computed relative free energies at room temperature (Δ*G*^0^ in cm^–1^) are also reported.

b Computed data are at the rDSD level (including LRA corrections for equilibrium rotational constants) except for electronic energies (junChSF12) and vibrational corrections to equilibrium rotational constants (B3).

c Standard errors are shown in parentheses in units of the last digits.

**Figure 7:**
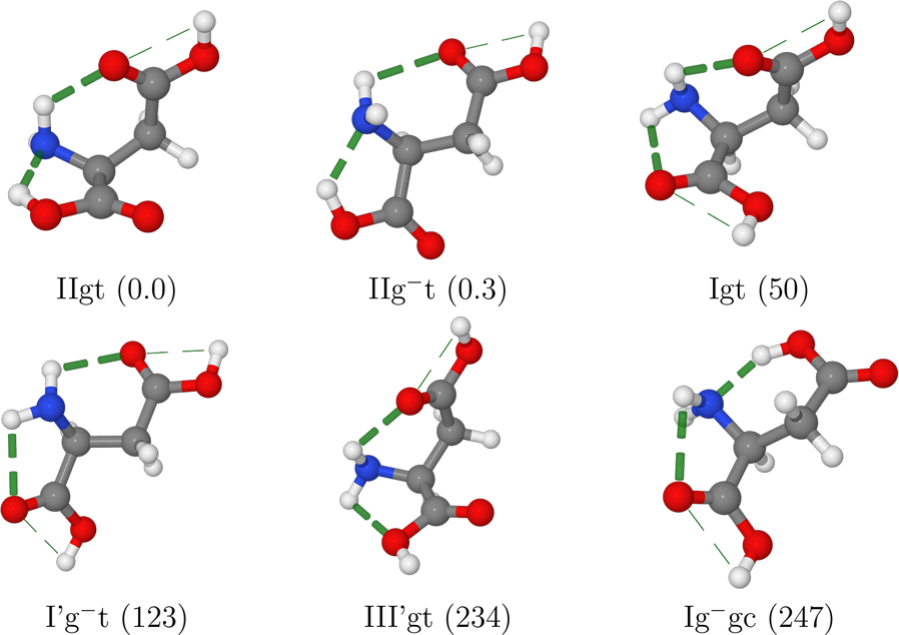
Conformers of aspartic acid detected in MW experiments with the computed relative free energies at room temperature (in cm^–1^) given in parentheses. H-bonds are highlighted by dashed lines.

Let us now analyze aspartic acid, the simplest amino acid containing two carboxylic groups. The CH_2_COOH side chain has three dihedral angles (χ_1_ = N–C^α^–C^β^–C^γ^, χ_2_ = C^α^–C^β^–C^γ^–O(H) and χ_3_ = C^β^–C^γ^–O–H). However, χ_3_ is frozen in trans (favored and not explicitly labeled in the following) or cis (labeled by c in the following) conformations. A recent systematic analysis of the conformational landscape^116^ identified 19 energy minima in a range of 3500 cm^–1^, and we were able to locate all those minima by our general exploration strategy with enlarged energy thresholds. Within this panel of candidates, only 9 conformers have electronic energies lying within 1000 cm^–1^ above the absolute energy minimum (see Table 11). Once again, a good quantitative agreement is observed between junChSF12 and rDSD results with the MAX and MUE between the two methods being 83.5 and 30.2 cm^–1^ without any inversion in the relative stability order. Inclusion of zero point and thermal effects produces significant changes in the trend issued from relative electronic energies with the most striking effect being, as usual, the destabilization of all the conformers showing type II hydrogen bridges (see Table 11). The six most populated conformers shown in Figure 7 are significantly more stable than the next 3 ones, and exactly six species were detected in MW experiments.^96^

**Table 11:** rDSD Relative Electronic Energies, Harmonic Zero Point Energies, Thermal Contributions and Quasi-harmonic Corrections, together with Difference with JunChSF12 Electronic Energies and B3 Anharmonic Corrections (all in cm^–1^) for the Low-Lying Conformers of Aspartic Acid^a^

Label	Δ*E*_*rDSD*_	ΔChS	ΔZPE_*H*_	ΔTh_*H*_	ΔZPE_(*anh*–*H*)_	*T*Δ*S*_(*QH*–*H*)_	Δ*G*°^b^	ϕ′	ψ	ω	χ_1_	χ_2_
IIgt	0.0	0.0	0.0	0.0	0.0	0.0	0.0	36.3	20.7	–6.3	61.7	168.8
IIg^–^t	133.5	7.5	–57.1	–60.8	–13.1	–9.7	0.3	–35.7	20.0	–4.3	–65.5	174.7
Igt	288.7	–4.3	–143.6	–80.1	–17.3	7.0	50.4	179.6	–164.5	–177.8	67.8	–177.2
Ig^–^gc	341.0	–4.1	7.3	–27.4	–38.8	–30.9	247.1	164.6	162.4	177.2	–63.0	38.8
III′gt	350.5	83.5	–114.2	–99.3	–13.7	27.3	234.1	177.0	24.4	178.1	65.9	–179.7
I′g^–^t	478.9	–40.6	–178.2	–121.6	–24.5	9.1	123.1	86.6	–167.5	–177.0	–63.9	169.8
I′gg^–^c	682.8	74.2	–4.4	18.0	–21.9	3.0	751.7	85.9	–179.1	–165.3	62.5	–36.9
IIItt	777.4	9.7	–86.0	–25.3	–12.7	–65.0	598.1	169.6	5.1	167.2	–158.3	171.1
I′tt	1136.3	–17.7	–202.4	–193.0	–17.3	51.1	757.0	62.7	–179.5	58.6	–173.7	–160.1

b Sum of columns 2, 3, 4, 5, 6, and 7.

a Best estimates of relative free energies at room temperature (Δ*G*° in cm^–1^) and dihedral angles optimized at the rDSD level (ϕ′, ψ, ω, χ_1_ = N–C^α^–C^β^–C^γ^, and χ_2_ = C^α^–C^β^–C^γ^–O(H) in degrees) are also given. The χ_3_ angle (C^β^–C^γ^–O–H) is always close to 180° (not explicitly indicated) or 0° (evidenced by the last “c” in the conformer label). See main text for details.

Both the I′g^–^t and III′gt conformers are more stable than their I and III counterparts due to the replacement of an intrabackbone bifurcated NH_2_···O=C or NH_2_···OH hydrogen bond by a single HNH···O=C or HNH···OH hydrogen bond plus a single HNH···O=C backbone (side chain) hydrogen bond. The increased stability explains also the absence of low-barrier relaxation paths from these conformers to I structures.

The spectroscopic parameters collected in Table 12 show a remarkable agreement between theory and experiment. It is noteworthy that previous MP2/6-311++G(d,p)^96^ computations forecasted that one or two different conformers should be experimentally detected and that the spectroscopic constants obtained at that level show MAX and MUE with respect to experiment (29.2 and 10.6 MHz) more than three times larger than their rDSD-LRA counterparts (8.2 and 3.1 MHz). The rDSD MUE (smaller than 0.2%) approaches again the accuracy of state-of-the-art composite methods for small semirigid molecules^117^ and permits the unbiased assignment of MW spectra.^118^ The stability order of the six most populated conformers is, however, quite different between theory and experimental estimates with the strongest discrepancy concerning the inversion of the relative stability of I and II species. Although the experimental populations take into account also possible relaxation of higher-energy structures to the most stable conformers, according to the computed free energies the initial populations of all the species outside the six most stable ones are too low to alter the computed relative populations. From another point of view, the experimental estimates are based on a number of assumptions, which might not be fulfilled in the present case. Also taking these considerations in mind, the agreement between theory and experiment concerning the nature and spectroscopic parameters of all the observable species remains remarkable.

**Table 12:** Ground-State Rotational Constants (*A*_0_, *B*_0_, and *C*_0_ in MHz), ^14^N-Nuclear Quadrupole Coupling Constants (χ in MHz), and Electric Dipole Moment Components (μ in debye) of the Six Most Stable Energy Minima of Aspartic Acid^a^

Conformer	IIgt	IIg^–^t	Igt	I′g^–^t	III′gt	Ig^–^gc
Computed^b^						
A_0_	2607.9	3412.3	2546.8	3372.8	2643.8	3192.2
B_0_	1188.9	900.4	1202.1	904.2	1182.9	943.8
C_0_	1057.1	762.5	1067.2	778.1	1055.9	781.4
χ_*aa*_	–3.7322	–3.4040	–0.2050	1.1611	–0.2629	–4.1388
χ_*bb*_	2.7326	1.4552	–0.2987	2.7491	–0.3570	2.5722
χ_*cc*_	0.9996	1.9488	0.5037	–3.9102	0.6199	1.5665
μ_*a*_	2.3532	3.6076	1.0967	0.5375	0.3702	–5.2042
μ_*b*_	4.1392	2.1025	1.2332	–1.8804	0.5037	1.1751
μ_*c*_	–2.1974	1.4410	1.7069	–0.7507	0.2090	–0.6972
Δ*G*^0^	0.0	0.3	50.4	123.1	234.1	247.1

Exp^c^	P	N	M	L	Q	O
A_0_	2612.20878(26)	3416.43489(66)	2553.85523(70)	3378.20873(26)	2651.953(31)	3198.861(19)
B_0_	1191.01132(17)	902.904474(79)	1205.08478(10)	907.373507(28)	1183.51697(30)	945.84803(7)
C_0_	1057.33169(16)	764.631177(96)	1069.14318(10)	780.042139(32)	1054.98929(34)	781.75139(18)
χ_*aa*_	–3.5601(63)	–3.3602(87)	–0.2774(35)	0.9560(35)	–0.295(27)	–3.995(19)
χ_*bb*_	2.6538(54)	1.4823(73)	–0.2640(35)	2.7296(23)	–0.350(45)	2.524(32)
χ_*cc*_	0.9064(54)	1.8778(73)	0.5414(35)	–3.6856(23)	0.645(45)	1.470(32)

a The computed relative free energies at room temperature (Δ*G*^0^ in cm^–1^) are also reported.

b Computed data are at the rDSD level (including LRA corrections for equilibrium rotational constants) except for electronic energies (junChSF12) and vibrational corrections to equilibrium rotational constants (B3).

c Standard errors are shown in parentheses in units of the last digits.

The last system considered in this study is asparagine, which is the only proteinogenic α-amino acid, together with glutamine,^119^ containing an amide group. The soft degrees of freedom of the asparagine side chain (CH_2_CONH_2_) include two dihedral angles (χ_1_ = N–C^α^–C^β^–C^γ^, χ_2_ = C^α^–C^β^–C^γ^–N) because the coupled rotation/inversion displacements of the NH_2_ amide moiety from the planar reference structure can be safely added to the panel of stiff degrees of freedom. The amide moiety can act either as a proton donor or as a proton acceptor, with this increasing the number of possible backbone (side chain) intramolecular hydrogen bonds. Asparagine in the gas-phase has been widely studied by both computational^47,120^ and experimental^47,121^ points of view, but a comprehensive characterization of its structure and conformational landscape has not yet been performed by state-of-the-art quantum chemical methods.

**Figure 8:**
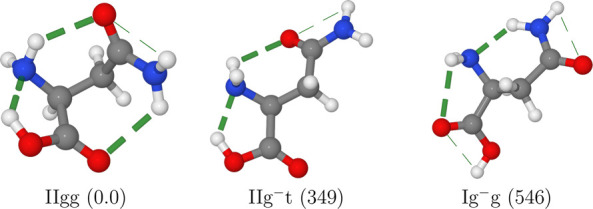
Most stable conformers of asparagine. The computed relative free energies at room temperature (in cm^–1^) are given in parentheses. H-bonds are highlighted by dashed lines.

The usual exploration/refinement strategy provides 5 conformers with rDSD electronic energies within a little more than 1000 cm^–1^ above the absolute energy minimum (see Table 13). At this level only the most stable IIgg conformer (see Figure 8) should be detectable in MW experiments. The situation is thus very different from that found in the case of aspartic acid because the presence of the NH_2_ amidic moiety in the side chain permits the compensation of the weak hydrogen bond in the carboxylic moiety (lacking in II structures of aspartic acid with respect to their I counterparts) by a backbone/side chain OH···NH_2_ hydrogen bond without reducing the local stability of the amide moiety. In fact, an analogous situation would involve a 180° rotation of the OH moiety in the carboxylic group of the side chain in aspartic acid away from its most stable arrangement.

**Table 13:** rDSD Relative Electronic Energies, Harmonic Zero Point Energies, Thermal Contributions, and Quasi-harmonic Corrections, together with Difference with JunChSF12 Electronic Energies and B3 Anharmonic Corrections (all in cm^–1^) for the Low-Lying Conformers of Asparagine^a^

Label	Δ*E*_*rDSD*_	ΔChS	ΔZPE_*H*_	ΔTh_*H*_	ΔZPE_(*anh*–*H*)_	*T*Δ*S*_(*QH*–*H*)_	Δ*G*°^b^	ϕ′	ψ	ω	χ_1_	χ_2_
IIgg	0.0	0.0	0.0	0.0	0.0	0.0	0.0	–23.3	15.4	–4.9	58.5	101.0
IIg^–^t	727.6	–24.2	–222.8	–193.4	16.6	44.8	348.6	–36.9	20.5	–4.3	–65.6	177.0
Ig^–^g	826.9	27.3	–212.0	–260.1	81.8	82.5	546.4	172.4	161.0	177.3	–69.6	34.9
I′gg^–^	1016.6	8.8	–198.6	–62.1	61.6	24.1	850.4	80.6	–164.8	–178.6	69.8	–29.5
Igt	1072.6	–36.1	–367.2	–271.5	154.4	66.3	568.5	–179.7	–164.6	–178.0	67.1	–173.1

a Best estimates of Gibbs free energies (Δ*G*° in cm^–1^) and dihedral angles optimized at the rDSD level (ϕ′, ψ, ω, χ_1_ = N–C^α^–C^β^–C^γ^, and χ_2_ = C^α^–C^β^–C^γ^–N in degrees) are also given. The χ_3_ angle (C^β^–C^γ^–N–H) is always close to 0°. See main text for details.

b Sum of columns 2, 3, 4, 5, 6, and 7.

ZPE and thermal contributions strongly stabilize all conformers with respect to the most stable IIgg strucure, so that the IIg^–^t form (see Figure 8) might become accessible to experimental characterization. As a matter of fact, several searches of transition states connecting IIg^–^t and IIgg conformers gave quite high energy barriers preventing any effective relaxation path. As a consequence, there is a disagreement between theory and experiment^47^ about the number of low-lying conformers of asparagine. However, comparison between computed and experimental spectroscopic parameters for the single conformer detected in the MW study of ref (47) shows the usual remarkable agreement (see Table 14) with MAX and MUE as low as 12.6 and 4.7 MHz for rotational constants and 0.19 and 0.08 MHz for quadrupole coupling constants.

**Table 14:** Experimental^47^ and Computed Ground State Rotational Constants (*A*_0_, *B*_0_, *C*_0_ in MHz) and Quadrupole Coupling Constants (χ in MHz) for the IIgg Conformer of Asparagine^a^

Experimental				Computed		
	*A*_0_	*B*_0_	*C*_0_	*A*_0_	*B*_0_	*C*_0_
	2270.85145(85)	1387.80238(41)	1102.63540(41)	2258.22	1387.0	1101.81

Experimental				Computed		
	χ_*aa*_	χ_*bb*_	χ_*cc*_	χ_*aa*_	χ_*bb*_	χ_*cc*_
N^α^	–2.0313(50)	2.5720(57)	–0.5408(57)	–2.2164	2.6624	–0.4459
N^β^	–1.4649(63)	1.5518(76)	–0.0870(76)	–1.5141	1.5607	–0.0466

a The values in parentheses are the experimental standard errors in units of the last digit.

### 5.3 Trends of Intramolecular Interactions

The accurate results obtained for several amino acids permit the strengths of the main interactions governing the conformational landscapes of these flexible systems to be estimated. In particular, approximate values for the strengths of different hydrogen bonds can be computed from prototypical systems and used to rationalize energy differences among the conformers of different amino acids in terms of sums of stabilizations from near-atom interactions. Based on the energy difference between Ic and I or I′c and I′ conformers of glycine, for each carboxyl group ω = 180° is more stable than ω = 0° by about 1700 cm^–1^ and the same applies to χ_3_ in the case of aspartic acid. Concerning other situations, the hydrogen bond donors can be ranked in the order O–H > N–H > S–H, and the hydrogen bond acceptors in the order *N* > O > S. As a consequence, the strongest hydrogen bond is H_2_N···H–O (with an estimated strength of 3200 cm^–1^), which involves the best donor and the best acceptor, followed by H_2_–N···HNH (with an estimated strength of 2400 cm^–1^). Those values, together with the energy differences among conformers I, II, and III of glycine, permit strengths of about of 1700 and 1100 cm^–1^ to be estimated for the bifurcated NH_2_···O=C and NH_2_···O–H hydrogen bonds. Furthermore, the difference between the pairs I, I′ and III, III′ leads to hydrogen bond strengths of about 1300 and 800 cm^–1^ for the more conventional single H–N–H···O=C and HNH···O–H hydrogen bonds. Finally, a comparable strength of about 800 cm^–1^ is estimated for the H_2_N···H–S, H–S···H–N–H, and S–H···O=C hydrogen bonds. It is then quite straightforward to understand why conformer I is more stable than its II counterpart in the absence of backbone (side chain) hydrogen bonds (e.g., in alanine): in fact, the sum of NH_2_···O=C and favorable carboxyl conformation exceeds by about 200 cm^–1^ the stronger H_2_N···H–O hydrogen bond but with an unfavorable conformation of the carboxylic moiety. On the other hand, in serine and threonine, conformer II becomes more stable due to the extra stabilization related to an O–H···O=C hydrogen bond involving the backbone and the side chain. The same occurs in cysteine, where the 800 cm^–1^ gained from the S–H···O=C hydrogen bond makes the IIgg conformer more stable than the Igg counterpart by about 600 cm^–1^. An analogous situation is found in aspartic acid, where the amine moiety is involved at the same time in an OH···N hydrogen bond within the backbone and a HNH···O=C hydrogen bond with the side chain. Finally, IIgg is by far the most stable conformer in asparagine because the presence of an amide group allows the formation of two additional backbone (side chain) hydrogen bonds. Type III conformers are intrinsically less stable than their I counterparts (due to the lower strength of NH_2_···O–H with respect to NH_2_···O=C hydrogen bond), and moreover, they can easily relax to I forms through rotation around ψ when not locked by additional interactions. However, III′ conformers featuring a single H–N–H···O–H hydrogen bond can be stabilized and locked into sufficiently deep energy wells upon involvement of the released N–H bond into additional hydrogen bonds with the side chain. This is the case, for instance, of the III′gg conformer in serine, threonine (H–O···H–N–H···O–H), and cysteine (H–O···H–N–H···S–H).

Hydrogen bonding is surely the driving force ruling the general trends of structures and relative stabilities, but the detailed geometry and energy changes between conformers depend strongly on other stereoelectronic effects like, e.g., hyperconjugation or steric repulsion. For instance, any additive picture based on individual hydrogen bond strengths is tuned by the preference of bulky vicinal substituents for trans or gauche conformations, which, in turn, depends on the balance between electrostatic, steric, and hyperconjugative effects. Furthermore, vibrational effects (affecting both ZPEs and entropic contributions) alter the stability order provided by relative electronic energies and must be taken into the proper account.

While the reader is referred to studies of specific systems for more detailed analyses along these lines,^46,47,96,111,115^ we point out that only the availability of accurate results including all the stereoelectronic and vibrational effects (like those reported in the present paper) can provide an unbiased reference for building more realistic models (e.g., force fields including non additive terms) for the study of flexible biomolecules.

## 6 Concluding Remarks

In this paper, a general strategy aimed at the unbiased disentanglement of the conformational bath of flexible biomolecule building blocks in the gas phase has been further improved and validated for the specific case of representative natural α-amino acids. The use of curvilinear internal coordinates permits the separation between stiff and soft degrees of freedom. Then, effective exploration of the soft variables can be performed by purposely tailored evolutionary algorithms, whose fitness scores are obtained by constrained geometry optimizations of the stiff degrees of freedom employing a fast semiempirical method. Refinement of the energies and structures by a hybrid and then a last-generation double-hybrid functional allows very reliable results to be obtained minimizing the number of expensive computations. Application of the procedure to supersonic jet experiments requires also the location of transition states ruling the interconversion between pairs of adjacent energy minima and the identification of fast relaxation processes. Improved structures and relative energies are obtained by the rDSD-LRA approach and the junChSF12 composite method, respectively. Finally, the spectroscopic parameters of sufficiently populated conformers can be safely computed at the rDSD level.

The results obtained for glycine, alanine, and, especially, different natural α-amino acids with polar side chains are in full agreement with the available spectroscopic data and permit their unbiased interpretation in terms of the cooperation or competition between intrabackbone and backbone (side chain) hydrogen bonds.

Together with the intrinsic interest of the studied molecules, the results of the present investigation show that highly reliable analysis of the conformational landscape is today possible for flexible building blocks of biomolecules in the gas phase. Furthermore, we provide benchmark results for the validation of cheaper quantum chemical methods, which become unavoidable for large biomolecules.

## Supporting Information Available

Full set of parameters of the IM-EA algorithm (Table S1) and different contributions to the relative energies and rotational constants of low-energy conformers for glycine (Table S2), alanine (Table S3), serine (Tables S4 and S5), threonine (Tables S6 and S7), cysteine (Tables S8 and S9), aspartic acid (Tables S10 and S11) and asparagine (Tables S12 and S13) (PDF)
