# Supplementary material for: Benchmark Structures and Conformational Landscapes of Amino Acids in the Gas Phase: A Joint Venture of Machine Learning, Quantum Chemistry, and Rotational Spectroscopy
Source: J Chem Theory Comput. 2023 Feb 2;19(4):1243–60. doi: 10.1021/acs.jctc.2c01143 (PMC9979611; doi:10.1021/acs.jctc.2c01143)
Supplement: Supplementary file 1 — ct2c01143_si_001.pdf [file ct2c01143_si_001.pdf]

Supporting Information: Benchmark structures and conformational landscapes of amino acids in the gas-phase: a joint venture of Machine Learning, Quantum Chemistry and Rotational Spectroscopy

Vincenzo Barone,*,† Marco Fusè,‡ Federico Lazzari,† and Giordano Mancini†

†*Scuola Normale Superiore di Pisa, piazza dei Cavalieri 7, 56126 Pisa, Italy*

‡*DMMT-sede Europa, Università di Brescia, viale Europa 11, 25121 Brescia, Italy*

E-mail: vincenzo.barone@sns.it

0.1 Exploration of the conformational potential energy surface (PES)

The full set of parameters selected for the IM-EA algorithm described in the main text are given in Table S1.

Table S1: Run parameters and values for PES exploration.

Parameter	Value
Population size	100
Number of generations (max)	50
Selection pressure	0.5
Selection method	Tournament
Tournament size	2
Elitism (last 10% generations)	T
Crossover method	SBX
Crossover probability	0.5
Mutation rate (parents)	0.3
Mutation rate (children)	0.5
Number of islands	4
Migration frequency	4
Migration size	0.05

1 Structure and energetics

1.1 Glycine and alanine.

Table S2: Relative energy for the low energy conformers of glycine in cm^{-1} (1 kJ/mol = 83.59 cm^{-1}) computed at the corresponding rDSD optimized geometries. All the computational levels include the F12 ansatz. CBS and CV contributions are evaluated the the MP2F12 level. The optimized dihedral angles (in degrees) are also given.

Label	CC/TZ	MP2/TZ	MP2/QZ	CBS	CBS+CV	ϕ'	ψ	ω
I	0.0	0.0	0.0	0.0	0.0	180.0	180.0	180.0
II*	229.9	125.8	134.2	244.5	236.5	-22.5	11.8	-2.1
I'*	460.4	429.5	431.2	463.3	461.5	83.1	-162.6	-177.0
III	600.6	575.4	576.1	601.8	605.8	180.0	0.0	180.0
III'*	923.8	907.7	911.8	930.9	935.3	60.7	38.3	178.4
Ic	1690.3	1734.0	1728.2	1680.3	1688.7	180.0	180.0	0.0
IIIc	2054.0	2108.9	2115.9	2062.0	2051.5	180.0	0.0	0.0
I'c	2175.5	2190.9	2188.9	2172.0	2176.9	93.2	-165.0	4.1

Table S3: Relative energy for the low energy conformers of alanine (in cm^{-1}) computed at the corresponding rDSD optimized geometries except for B3 computations, which employ B3 geometries. All the post-HF computational levels include the F12 ansatz. CBS and CV contributions are evaluated the the MP2F12 level. The optimized dihedral angles (in degrees) are also given.

Label	B3	CC/TZ	MP2/TZ	MP2/QZ	CBS	CBS+CV	ϕ'	ψ	ω
I	0.0	0.0	0.0	0.0	0.0	0.0	176.0	162.7	177.8
II^-	32.5	39.4	-60.9	-55.8	48.4	35.6	29.9	-16.7	3.7
II	-0.5	105.9	64.6	69.3	114.1	103.1	26.2	14.4	3.1
III^-	405.1	434.9	414.5	412.1	430.7	432.6	179,9	-42.2	-177.5
III	/[a]	432.5	433.5	433.6	432.7	436.0	-178.6	84.1	-175.7
I'	485.9	398.1	382.0	382.5	399.0	396.5	-77.4	153.9	176.4
I'^-	544.4	453.4	445.6	443.7	450.1	446.6	82.2	-167.1	-177.1
III'	722.7	628.9	651.4	643.2	614.7	613.5	61.8	49.4	175.5
III'^-	813.2	788.2	792.2	791.6	787.3	789.7	-58.1	-43.8	-178.1
Ic	1877.8	1739.8	1787.1	1780.5	1728.5	1736.0	176.4	162.0	-2.7
III^-c	2164.9	1996.3	1970.1	1965.5	1988.3	1980.5	-170.6	-13.8	3.1
I'c	2338.8	2122.6	2153.1	2147.6	2113.0	2116.5	-84.1	153.5	-6.4
I'^-c	2370.1	2160.8	2201.3	2195.2	2151.2	2154.9	74.2	-161.3	5.4

(a) relxes to conformer I.

1.2 Serine.

Table S4: Relative energy for the low energy conformers of serine (in cm^{-1}) computed at the corresponding rDSD optimized geometries. All the computational levels include the F12 ansatz. CBS and CV contributions are evaluated the the MP2F12 level.

Label	CC/TZ	MP2/TZ	MP2/QZ	CBS	CBS+CV
IIgg	0.0	0.0	0.0	0.0	0.0
Ig$^-$g	175.4	188.8	183.8	166.8	173.0
IItg$^-$	219.7	268.3	273.9	229.3	233.6
I'gg$^-$	306.9	297.3	289.5	293.5	294.6
III'gg	522.3	609.2	607.2	518.8	531.5
IIg$^-$t	636.0	562.5	562.1	635.4	634.6
III'tg$^-$	795.6	904.7	899.7	787.1	800.9
IIg$^-$g$^-$	649.5	596.2	595.8	648.8	650.9
IIIg$^-$g	768.5	762.9	755.8	756.2	766.0
IIg$^-$t	793.2	805.1	804.5	792.0	794.4
Igt	860.3	922.8	916.3	849.0	862.4
Igg	855.8	878.8	874.2	847.9	857.6

Table S5: Equilibrium rotational constants of low-energy serine conformers obtained at the rDSD level (rDSD) and also including linear regression corrections (rDSD-LRA) together with vibrational corrections at the B3 level of theory. All the values are in MHz.

	rDSD			rDSD-LRA			B3		
	A_e	B_e	C_e	A_e	B_e	C_e	$\Delta_{vib}A$	$\Delta_{vib}B$	$\Delta_{vib}C$
IIgg	3559.25	2392.98	1739.74	3578.05	2404.66	1748.15	28.72	32.28	13.48
Ig$^-$g	4487.04	1822.69	1451.78	4510.37	1831.61	1459.00	49.03	8.60	17.05
IItg$^-$	3646.33	2394.21	1521.56	3664.23	2406.03	1529.05	33.37	23.51	13.77
I'gg$^-$	3540.65	2313.08	1801.99	3559.68	2324.21	1810.59	53.94	19.00	6.97
III'gg	3971.09	2229.17	1670.13	3991.88	2239.94	1678.27	41.56	17.03	21.24
IIg$^-$t	4531.44	1846.89	1471.70	4554.88	1856.03	1479.06	46.75	13.03	17.01
III'tg$^-$	3492.43	2327.53	1599.58	3510.42	2338.85	1607.34	45.58	34.17	2.60
IIg$^-$g$^-$	4487.75	1831.07	1468.13	4510.98	1840.04	1475.42	44.81	12.42	16.64
IIIg$^-$g	4525.20	1773.44	1483.36	4548.81	1782.14	1490.71	43.67	9.30	17.59
IIg$^-$t	4647.50	1862.01	1388.38	4671.64	1871.21	1395.35	44.8	15.54	10.53
Igt	3476.16	2302.40	1809.01	3494.95	2313.62	1817.68	43.54	15.71	11.46
Igg	3455.81	2373.73	1743.12	3474.42	2385.12	1751.47	55.66	48.71	2.40

1.3 Threonine.

Table S6: Relative energy for the low energy conformers of threonine (in cm^{-1}) computed at the corresponding rDSD optimized geometries. All the computational levels include the F12 ansatz. CBS and CV contributions are evaluated the the MP2F12 level.

Label	CC/TZ	MP2/TZ	MP2/QZ	CBS	CBS+CV
IIgg	0.0	0.0	0.0	0.0	0.0
Ig$^-$g	251.7	227.0	222.1	243.2	253.0
IItg$^-$	363.7	378.6	382.3	370.2	374.3
I'gg$^-$	431.8	422.5	415.9	420.4	423.4
III'g$^-$g	622.3	591.9	585.6	611.4	620.4
III'gg	621.5	706.1	705.5	620.6	635.0
IIg$^-$t	723.1	648.0	644.4	716.9	720.4
IIgt	596.8	579.3	578.1	594.8	593.0
IIg$^-$g$^-$	747.4	691.6	689.9	744.4	750.3
Igt	965.1	1006.0	1002.7	959.5	971.1

Table S7: Equilibrium rotational constants of threonine obtained at the rDSD level (rDSD) and also including linear regression corrections (rDSD-LRA) together with vibrational corrections at the B3 level of theory. All the values are in MHz.

	rDSD			rDSD-LRA			B3		
	A_e	B_e	C_e	A_e	B_e	C_e	$\Delta_{vib}A$	$\Delta_{vib}B$	$\Delta_{vib}C$
IIgg	3240.70	1544.01	1266.99	3256.98	1550.59	1272.42	33.31	22.25	7.31
Ig$^-$g	2877.72	1608.89	1218.91	2891.05	1616.20	1224.32	26.57	13.98	9.55
IItg$^-$	2689.30	1782.85	1379.71	2701.62	1790.53	1385.92	29.74	15.77	9.16
I'gg$^-$	3158.19	1508.03	1319.49	3174.26	1514.38	1325.06	32.68	12.99	11.91
III'g$^-$g	2897.30	1571.58	1247.29	2910.71	1578.74	1252.75	25.04	13.88	8.90
III'gg	3388.93	1480.88	1242.76	3405.95	1487.16	1248.17	30.19	12.44	13.48
IIg$^-$t	2918.60	1665.54	1194.04	2931.98	1672.98	1199.36	24.36	16.58	11.74
IIgt	3186.72	1490.06	1290.20	3202.68	1496.36	1295.66	32.22	9.37	10.75
IIg$^-$g$^-$	2914.85	1650.33	1191.60	2928.33	1657.75	1196.94	24.65	14.53	10.90
Igt	3144.67	1505.26	1317.40	3160.70	1511.68	1322.99	33.08	11.77	10.37
IIIgt	3183.19	1485.80	1320.75	3199.44	1492.11	1326.32	29.13	11.86	13.29

1.4 Cysteine.

Table S8: Relative energy for the low-lying conformers of cysteine (in cm^{-1}) computed at the corresponding rDSD optimized geometries. All the computational levels include the F12 ansatz. CBS and CV contributions are evaluated the the MP2F12 level.

Label	CC/TZ	MP2/TZ	MP2/QZ	CBS	CBS+CV
IIgg	0.0	0.0	0.0	0.0	0.0
IIg$^-$g$^-$	527.9	545.8	546.7	529.5	537.4
Igg	557.5	648.2	644.6	551.3	561.8
Ig$^-$g	622.6	731.7	731.5	622.4	639.6
III'gg	690.2	786.0	777.1	674.8	688.2
III'tg$^-$	897.4	1007.9	1005.0	892.4	912.8
I'gg$^-$	681.2	707.6	704.7	676.1	678.2
III'gg$^-$	989.4	1016.8	1014.9	986.1	991.2
IIgt	1040.5	1158.5	1158.1	1039.8	1056.0

Table S9: Equilibrium rotational constants for low-energy conformers of cysteine obtained at the rDSD level (rDSD) and also including linear regression corrections (rDSD-LRA) together with vibrational corrections at the B3 level of theory. All the values are in MHz.

	rDSD			rDSD-LRA			B3		
	A_e	B_e	C_e	A_e	B_e	C_e	$\Delta_{vib}A$	$\Delta_{vib}B$	$\Delta_{vib}C$
IIgg	3069.41	1607.14	1330.06	3086.31	1617.08	1337.75	23.04	16.49	10.41
IIg$^-$g$^-$	4366.04	1175.40	1016.39	4388.20	1182.81	1022.65	35.86	9.10	9.91
Igg	2889.34	1623.67	1363.66	2905.54	1633.61	1371.52	31.10	18.01	4.57
Ig$^-$g	4224.37	1181.49	1006.27	4246.24	1188.88	1012.41	28.67	7.09	11.59
IIIgg	3205.73	1575.03	1276.89	3223.23	1584.73	1284.28	0.10	21.56	16.78
III'tg$^-$	2976.29	1534.56	1217.94	2992.01	1544.22	1225.15	2.48	19.92	15.03
I'gg$^-$	2853.71	1683.78	1402.22	2869.68	1693.91	1410.16	37.73	11.50	9.01
III'gg$^-$	4386.66	1185.94	1015.51	4408.98	1193.41	1021.76	29.34	7.12	12.69
IIgt	3522.78	1447.90	1082.24	3540.12	1457.17	1088.72	27.33	13.14	9.62

1.5 Aspartic Acid.

Table S10: Relative energy for the low-lying conformers of aspartic acid (in cm^{-1}) computed at the corresponding rDSD optimized geometries. All the computational levels include the F12 ansatz. CBS and CV contributions are evaluated the the MP2F12 level.The optimized χ_3 dihedral angle (in degrees) is also given.

Label	CC/TZ	MP2/TZ	MP2/QZ	CBS	CBS+CV	χ_3
IIgt	0.0	0.0	0.0	0.0	0.0	179.7
IIg$^-$t	141.5	180.1	180.1	141.6	141.0	179.0
Igt	276.7	321.3	318.7	272.3	284.4	-179.8
Ig$^-$gc	334.4	353.6	368.2	324.5	336.9	-3.1
IIIgt	400.8	394.5	388.8	426.1	434.0	-179.7
I'g$^-$t	443.0	478.4	473.3	434.2	438.3	179.9
I'gg$^-$c	732.6	619.6	632.6	755.1	757.0	1.1
IIItt	778.4	900.0	898.5	775.9	787.1	179.4
I'tt	974.1	938.1	939.0	975.7	977.2	-178.7

Table S11: Equilibrium rotational constants for the six observed conformers of aspartic acid obtained at the rDSD level (rDSD) and also including linear regression corrections (rDSD-LRA) together with vibrational corrections at the B3 level of theory. All the values are in MHz.

		rDSD			rDSD-LRA			B3		
		A_e	B_e	C_e	A_e	B_e	C_e	$\Delta_{vib}A$	$\Delta_{vib}B$	$\Delta_{vib}C$
IIgt	2605.13	1196.59	1060.03	2617.41	1201.79	1064.66	9.52	12.90	7.54	
IIg$^-$t	3423.02	903.91	765.37	3440.58	907.74	768.71	28.31	7.34	6.16	
Igt	2561.68	1204.47	1069.77	2573.99	1209.72	1074.41	27.18	7.66	7.17	
Ig$^-$gc	3199.45	945.23	784.94	3215.86	949.14	788.33	23.65	5.37	6.96	
IIIgt	2645.31	1185.25	1058.96	2657.78	1190.46	1063.57	13.85	7.60	7.62	
I'g$^-$t	3398.73	907.73	779.87	3416.51	911.54	783.23	43.75	7.38	5.09	

1.6 Asparagine.

Table S12: Relative energy for the low-lying conformers of asparagine (in cm^{-1}) computed at the corresponding rDSD optimized geometries. All the computational levels include the F12 ansatz. CBS and CV contributions are evaluated the the MP2F12 level.

Label	CC/TZ	MP2/TZ	MP2/QZ	CBS	CBS+CV
IIgg	0.0	0.0	0.0	0.0	0.0
IIg$^-$t	709.9	878.0	875.1	704.8	703.4
Ig$^-$g	836.3	947.3	949.9	840.9	854.2
I'gg$^-$	1015.4	1049.2	1051.0	1018.4	1025.4
Igt	1032.5	1191.5	1188.0	1026.5	1036.5

Table S13: Equilibrium rotational constants for low-energy conformers of asparagine obtained at the rDSD level (rDSD) and also including linear regression corrections (rDSD-LRA) together with vibrational corrections at the B3DZ level of theory. All the values are in MHz.

	rDSD			rDSD-LRA			B3		
	A_e	B_e	C_e	A_e	B_e	C_e	$\Delta_{vib}A$	$\Delta_{vib}B$	$\Delta_{vib}C$
IIgg	2267.07	1396.48	1105.36	2277.72	1402.34	1110.09	19.50	15.34	8.28
IIg$^-$t	3397.37	912.81	766.93	3414.27	916.57	770.19	32.44	6.82	7.18
Ig$^-$g	3083.86	940.69	781.96	3099.24	944.58	785.30	25.16	4.96	7.62
I'gg$^-$	2349.94	1284.21	1105.70	2360.92	1289.63	1110.50	41.29	0.31	3.94
Igt	2573.23	1210.53	1078.30	2585.61	1215.59	1082.81	37.96	4.16	8.13
